# Supplementary material for: Kelpie: generating full-length ‘amplicons’ from whole-metagenome datasets
Source: PeerJ. 2019 Jan 30;6:e6174. doi: 10.7717/peerj.6174 (PMC6359901; doi:10.7717/peerj.6174)
Supplement: Table S2 — Full OTU table produced from both amplicon and Kelpie-generated sequences for all three coal seam microbiome samples, ordered by total abundance. Red amplicon counts indicated that mapping the WGS reads for the sample back to the consensus sequence for the OTU showed that it had less than 90% kMer coverage. The green amplicon counts show where the WGS reads for a sample gave 100% coverage of the OTU sequence. This table is derived from the ‘AE’ tabs in the Excel spreadsheet ‘Kelpie - CSM.xlsx’ which is available as Table S6. [file peerj-07-6174-s002.docx]

*Table S2: Full OTU table produced from both* ***amplicon and Kelpie-generated sequences*** *for all three coal seam microbiome samples, ordered by total abundance. Red amplicon counts indicated that mapping the WGS reads for the sample back to the consensus sequence for the OTU showed that it had less than 90% kMer coverage. The green amplicon counts show where the WGS reads for a sample gave 100% coverage of the OTU sequence. This table is derived from the ‘AE’ tabs in the Excel spreadsheet ‘Kelpie - CSM.xlsx’ which is available as Supplemental Table S6.*

| OTU | Size | CSM Species | W1 amplicon | W1 extended | W2 amplicon | W2 extended | W3 amplicon | W3 extended | amplicon reads % | cumulative |
| --- | --- | --- | --- | --- | --- | --- | --- | --- | --- | --- |
| 1 | 43603 | Desulfuromonas acetexigens (T) (U23140) | 27333 | 13574 | 132 | 0 | 1554 | 1010 | 23.91% | 23.9% |
| 2 | 24970 | Thermodesulfovibrio aggregans (T) TGE-P1 (AB021302) | 24 | 0 | 17120 | 7816 | 10 | 0 | 13.69% | 37.6% |
| 3 | 10514 | Treponema zuelzerae (T) type strain: DSM 1903; 2 (FR749929) | 13 | 0 | 1171 | 305 | 5956 | 3069 | 5.77% | 43.4% |
| 4 | 10163 | Methanobacterium subterraneum (T) A8p, DSM 11074 (X99044) | 5 | 0 | 29 | 0 | 7736 | 2393 | 5.57% | 48.9% |
| 5 | 7081 | Cytophaga fermentans (T) ATCC 19072 (M58766) | 9 | 0 | 5845 | 1220 | 7 | 0 | 3.88% | 52.8% |
| 7 | 6514 | Methanosaeta harundinacea (T) 8Ac (AY817738) | 1032 | 192 | 16 | 0 | 3332 | 1942 | 3.57% | 56.4% |
| 6 | 6264 | Parabacteroides distasonis (T) JCM 5825 (AB238922) | 1270 | 271 | 9 | 0 | 3116 | 1598 | 3.44% | 59.8% |
| 8 | 5520 | Thermacetogenium phaeum (T) PB (AB020336) | 5 | 0 | 14 | 0 | 3285 | 2216 | 3.03% | 62.9% |
| 10 | 4837 | candidate division OP1 clone OPB14 (AF027045) | 3 | 0 | 4057 | 771 | 6 | 0 | 2.65% | 65.5% |
| 12 | 4611 | Lysinibacillus sp. LAM612 (KF443809) | 3 | 0 | 7 | 0 | 533 | 4068 | 2.53% | 68.0% |
| 9 | 4258 | Methanosarcina siciliae type strain: DSM3028 (FR733698) | 1238 | 2733 | 11 | 0 | 54 | 222 | 2.34% | 70.4% |
| 13 | 3847 | Methanocalculus pumilus (T) MHT-1 (AB008853) | 3312 | 476 | 30 | 0 | 29 | 0 | 2.11% | 72.5% |
| 11 | 3652 | Desulfotomaculum acetoxidans (T) DSM 771 (Y11566) | 6 | 0 | 2463 | 1177 | 6 | 0 | 2.00% | 74.5% |
| 14 | 3390 | Syntrophaceticus schinkii (T) Sp3 (EU386162) | 6 | 0 | 2871 | 506 | 7 | 0 | 1.86% | 76.3% |
| 15 | 3383 | Methanobacterium aarhusense (T) H2-LR (AY386124) | 1 | 0 | 3104 | 271 | 7 | 0 | 1.86% | 78.2% |
| 17 | 3012 | Methanothermobacter thermoflexus (T) IDZ, VKM B-1963, DSM 7268 (X99047) | 1 | 0 | 2685 | 326 | 0 | 0 | 1.65% | 79.9% |
| 16 | 2920 | Sulfurospirillum alkalitolerans HTRB-L1 (GQ863490) | 2340 | 508 | 41 | 0 | 31 | 0 | 1.60% | 81.5% |
| 21 | 2114 | Methanobacterium alcaliphilum (T) NBRC 105226 (AB496639) | 2 | 0 | 1161 | 100 | 586 | 265 | 1.16% | 82.6% |
| 18 | 2099 | Clostridium hungatei (T) AD; ATCC 700212 (AF020429) | 5 | 0 | 5 | 0 | 1124 | 965 | 1.15% | 83.8% |
| 20 | 2067 | Natronincola peptidivorans (T) Z-7031 (EF382661) | 12 | 0 | 8 | 0 | 1293 | 754 | 1.13% | 84.9% |
| 19 | 1955 | Pontibacter sp. JC215 A10 (HG008901) | 4 | 0 | 2 | 0 | 931 | 1018 | 1.07% | 86.0% |
| 23 | 1734 | Porphyromonas pogonae strain MI 10-1288 (NR 136443.1) | 1059 | 128 | 29 | 0 | 389 | 129 | 0.95% | 86.9% |
| 25 | 1557 | Acetobacterium malicum (T) DSM 4132 (X96957) | 929 | 304 | 16 | 0 | 153 | 155 | 0.85% | 87.8% |
| 22 | 1515 | Desulfovibrio oxamicus (T) DSM 1925 (DQ122124) | 19 | 10 | 2 | 0 | 860 | 624 | 0.83% | 88.6% |
| 24 | 1513 | Unclassified Bacteria | 2 | 0 | 955 | 556 | 0 | 0 | 0.83% | 89.4% |
| 26 | 1507 | Ignavibacterium album (T) Mat9-16 (AB478415) | 1 | 0 | 1089 | 408 | 9 | 0 | 0.83% | 90.3% |
| 28 | 1165 | Thermodesulfovibrio aggregans (T) TGE-P1 (AB021302) | 2 | 0 | 722 | 440 | 1 | 0 | 0.64% | 90.9% |
| 32 | 1055 | Ornatilinea apprima P3M-1 (JQ292916) | 796 | 254 | 3 | 0 | 2 | 0 | 0.58% | 91.5% |
| 27 | 977 | Moorella humiferrea (T) 64 FGQ (GQ872425) | 1 | 0 | 129 | 847 | 0 | 0 | 0.54% | 92.0% |
| 29 | 844 | Caldicoprobacter guelmensis (T) D2C22 (JQ707908) | 17 | 0 | 7 | 0 | 477 | 343 | 0.46% | 92.5% |
| 30 | 694 | Syntrophobacter sulfatireducens (T) TB8106 (AY651787) | 219 | 65 | 17 | 0 | 254 | 139 | 0.38% | 92.9% |
| 34 | 587 | Desulfovibrio alkalitolerans (T) RT2 (AY649785) | 108 | 23 | 4 | 0 | 429 | 23 | 0.32% | 93.2% |
| 31 | 586 | Magnetospira thiophila (T) MMS-1 (EU861390) | 6 | 0 | 5 | 0 | 290 | 285 | 0.32% | 93.5% |
| 33 | 566 | Sunxiuqinia faeciviva (T) JAM-BA0302 (AB362263) | 53 | 13 | 111 | 82 | 119 | 188 | 0.31% | 93.8% |
| 49 | 485 | Sporomusa ovata strain DSM 2662 (NR 117659.1) | 1 | 0 | 0 | 0 | 309 | 175 | 0.27% | 94.1% |
| 35 | 472 | Unclassified Bacteria | 0 | 0 | 259 | 211 | 2 | 0 | 0.26% | 94.3% |
| 36 | 407 | Thiohalocapsa marina (T) type strain: JA142 (AM491592) | 304 | 98 | 0 | 0 | 5 | 0 | 0.22% | 94.6% |
| 37 | 395 | Smithella propionica (T) LYP (AF126282) | 7 | 0 | 217 | 65 | 81 | 25 | 0.22% | 94.8% |
| 165 | 365 | Unclassified Bacteroidetes | 1 | 0 | 264 | 100 | 0 | 0 | 0.20% | 95.0% |
| 38 | 348 | Dethiosulfatibacter aminovorans (T) C/G2 (= JCM 13356, = NBRC 101112, = DSM 17477) (AB218661) | 230 | 115 | 0 | 0 | 3 | 0 | 0.19% | 95.2% |
| 54 | 346 | Dethiobacter alkaliphilus (T) AHT 1 (EF422412) | 4 | 0 | 43 | 84 | 116 | 99 | 0.19% | 95.4% |
| 39 | 292 | Aminiphilus circumscriptus (T) ILE-2 (AY642589) | 205 | 82 | 2 | 0 | 3 | 0 | 0.16% | 95.5% |
| 40 | 271 | Pseudomonas songnenensis strain NEAU-ST5-5 (NR 148295.1) | 6 | 0 | 1 | 0 | 138 | 126 | 0.15% | 95.7% |
| 41 | 265 | Desulfitobacterium metallireducens (T) 853-15A (AF297871) | 0 | 0 | 1 | 0 | 146 | 118 | 0.15% | 95.8% |
| 46 | 259 | Pelotomaculum propionicicum (T) MGP (AB154390) | 4 | 0 | 188 | 67 | 0 | 0 | 0.14% | 96.0% |
| 43 | 257 | Desulfitibacter alkalitolerans (T) sk.kt5 (AY538171) | 0 | 0 | 2 | 0 | 181 | 74 | 0.14% | 96.1% |
| 58 | 244 | Bellilinea caldifistulae (T) GOMI-1 (AB243672) | 2 | 0 | 93 | 48 | 51 | 50 | 0.13% | 96.2% |
| 45 | 232 | Unclassified Bacteria | 161 | 70 | 1 | 0 | 0 | 0 | 0.13% | 96.4% |
| 47 | 219 | Ruminococcaceae bacterium ZWB 4 (HG003571) | 169 | 47 | 2 | 0 | 1 | 0 | 0.12% | 96.5% |
| 50 | 212 | Olivibacter sitiensis (T) AW-6 (DQ421387) | 175 | 33 | 0 | 0 | 4 | 0 | 0.12% | 96.6% |
| 42 | 210 | Dielma fastidiosa strain JC13 (NR 125593.1) | 0 | 0 | 0 | 0 | 32 | 178 | 0.12% | 96.7% |
| 48 | 208 | Syntrophomonas bryantii type strain: DSM 3014 (HE654006) | 53 | 21 | 4 | 0 | 61 | 69 | 0.11% | 96.8% |
| 44 | 203 | Methanospirillum hungatei strain JF-1 (NR 074177.1) | 67 | 135 | 0 | 0 | 1 | 0 | 0.11% | 96.9% |
| 196 | 193 | Caldicoprobacter algeriensis TH7C1 (GU216701) | 0 | 0 | 133 | 60 | 0 | 0 | 0.11% | 97.0% |
| 53 | 192 | Marivirga sericea (T) IFO 15983 (AB078081) | 125 | 58 | 8 | 0 | 1 | 0 | 0.11% | 97.1% |
| 51 | 192 | Aminivibrio pyruvatiphilus 4F6E (AB623229) | 128 | 62 | 2 | 0 | 0 | 0 | 0.11% | 97.3% |
| 52 | 184 | Thermodesulfovibrio yellowstonii (T) YP87 (AB231858) | 0 | 0 | 107 | 77 | 0 | 0 | 0.10% | 97.4% |
| 166 | 175 | Smithella propionica (T) LYP (AF126282) | 84 | 33 | 4 | 0 | 29 | 25 | 0.10% | 97.4% |
| 62 | 175 | Syntrophorhabdus aromaticivorans (T) UI (AB212873) | 25 | 3 | 47 | 19 | 25 | 56 | 0.10% | 97.5% |
| 56 | 157 | Clostridium thermocellum (T) ATCC 27405 (CP000568) | 0 | 0 | 2 | 0 | 106 | 49 | 0.09% | 97.6% |
| 55 | 149 | Thermanaerothrix daxensis strain GNS-1 (NR 117865.1) | 0 | 0 | 70 | 79 | 0 | 0 | 0.08% | 97.7% |
| 57 | 148 | Ornatilinea apprima P3M-1 (JQ292916) | 109 | 34 | 2 | 0 | 3 | 0 | 0.08% | 97.8% |
| 61 | 125 | Syntrophorhabdus aromaticivorans (T) UI (AB212873) | 7 | 0 | 2 | 0 | 82 | 34 | 0.07% | 97.9% |
| 63 | 120 | Petrimonas sulfuriphila (T) BN3 (AY570690) | 104 | 16 | 0 | 0 | 0 | 0 | 0.07% | 97.9% |
| 64 | 118 | Geoalkalibacter ferrihydriticus (T) Z-0531 (DQ309326) | 4 | 0 | 2 | 0 | 61 | 51 | 0.06% | 98.0% |
| 59 | 118 | Spirochaeta smaragdinae (T) SEBR 4228; DSM 11293 (U80597) | 4 | 0 | 0 | 0 | 61 | 53 | 0.06% | 98.1% |
| 60 | 116 | Clostridium luticellarii strain FW431 (NR 145907.1) | 0 | 0 | 0 | 0 | 53 | 63 | 0.06% | 98.1% |
| 204 | 114 | Dethiobacter alkaliphilus (T) AHT 1 (EF422412) | 1 | 0 | 63 | 30 | 15 | 5 | 0.06% | 98.2% |
| 68 | 112 | Azoarcus olearius DQS-4 (EF158388) | 71 | 18 | 10 | 0 | 13 | 0 | 0.06% | 98.2% |
| 65 | 101 | Dethiobacter alkaliphilus (T) AHT 1 (EF422412) | 0 | 0 | 58 | 41 | 2 | 0 | 0.06% | 98.3% |
| 66 | 92 | Smithella propionica (T) LYP (AF126282) | 59 | 32 | 1 | 0 | 0 | 0 | 0.05% | 98.4% |
| 67 | 86 | Desulfotomaculum kuznetsovii strain 17 (NR 115129.1) | 0 | 0 | 50 | 35 | 1 | 0 | 0.05% | 98.4% |
| 76 | 83 | Smithella propionica (T) LYP (AF126282) | 54 | 24 | 3 | 0 | 2 | 0 | 0.05% | 98.4% |
| 71 | 79 | Atopobium vaginae (T) CCUG 38953 (Y17195) | 51 | 0 | 14 | 0 | 14 | 0 | 0.04% | 98.5% |
| 69 | 78 | Unclassified Clostridiales | 43 | 35 | 0 | 0 | 0 | 0 | 0.04% | 98.5% |
| 70 | 77 | Crocinitomix catalasitica (T) IFO 15977 (AB078042) | 38 | 37 | 2 | 0 | 0 | 0 | 0.04% | 98.6% |
| 72 | 75 | Ruminococcaceae bacterium ZWB 4 (HG003571) | 4 | 0 | 1 | 0 | 46 | 24 | 0.04% | 98.6% |
| 74 | 72 | uncultured bacterium KF-JG30-18 (AJ295656) | 0 | 0 | 44 | 28 | 0 | 0 | 0.04% | 98.7% |
| 73 | 72 | Tepidanaerobacter syntrophicus (T) JL (AB106353) | 0 | 0 | 0 | 0 | 32 | 40 | 0.04% | 98.7% |
| 75 | 66 | Leptolinea tardivitalis (T) YMTK-2 (AB109438) | 0 | 0 | 29 | 37 | 0 | 0 | 0.04% | 98.7% |
| 199 | 65 | Aminivibrio pyruvatiphilus 4F6E (AB623229) | 60 | 5 | 0 | 0 | 0 | 0 | 0.04% | 98.8% |
| 79 | 65 | Thermoanaerobacter pseudethanolicus ATCC 33223 (CP000924) | 0 | 0 | 6 | 0 | 33 | 26 | 0.04% | 98.8% |
| 83 | 61 | Methanobacterium formicicum (T) DSM 1535 (AF169245) | 10 | 0 | 18 | 0 | 33 | 0 | 0.03% | 98.8% |
| 78 | 60 | Veillonella magna (T) lac18 (EU096495) | 5 | 0 | 0 | 0 | 28 | 27 | 0.03% | 98.9% |
| 99 | 58 | Clostridium thermosuccinogenes (T) DSM 5807 (Y18180) | 11 | 6 | 0 | 0 | 29 | 12 | 0.03% | 98.9% |
| 80 | 58 | Longilinea arvoryzae (T) KOME-1 (AB243673) | 0 | 0 | 1 | 0 | 22 | 35 | 0.03% | 98.9% |
| 81 | 52 | Acidobacteria bacterium P105 (KJ461654) | 0 | 0 | 31 | 21 | 0 | 0 | 0.03% | 99.0% |
| 82 | 52 | Clostridium putrificum (T) DSM 1734 (X73442) | 23 | 23 | 4 | 0 | 2 | 0 | 0.03% | 99.0% |
| 85 | 52 | Desulfotomaculum varum RH04-3 (GU126374) | 2 | 0 | 0 | 0 | 39 | 11 | 0.03% | 99.0% |
| 77 | 51 | Unclassified Bacteria | 0 | 0 | 0 | 0 | 11 | 40 | 0.03% | 99.0% |
| 96 | 45 | Gracilibacter thermotolerans (T) JW/YJL-S1 (DQ117465) | 18 | 0 | 0 | 0 | 14 | 13 | 0.02% | 99.1% |
| 87 | 45 | Sphingomonas ginsenosidimutans (T) Gsoil 1429 (HM204925) | 0 | 0 | 0 | 0 | 0 | 45 | 0.02% | 99.1% |
| 100 | 44 | Proteiniphilum acetatigenes (T) TB107 (AY742226) | 27 | 6 | 4 | 0 | 7 | 0 | 0.02% | 99.1% |
| 93 | 42 | Olegusella massiliensis strain KHD7 (NR 146815.1) | 0 | 0 | 20 | 21 | 1 | 0 | 0.02% | 99.1% |
| 86 | 41 | Dethiobacter alkaliphilus (T) AHT 1 (EF422412) | 15 | 0 | 14 | 0 | 12 | 0 | 0.02% | 99.2% |
| 98 | 41 | Clostridium hungatei (T) AD; ATCC 700212 (AF020429) | 32 | 9 | 0 | 0 | 0 | 0 | 0.02% | 99.2% |
| 84 | 38 | Clostridium thermopalmarium (T) (X72869) | 0 | 0 | 0 | 0 | 17 | 21 | 0.02% | 99.2% |
| 102 | 37 | Desulfomicrobium salsuginis strain ADR21 (NR 132593.1) | 25 | 8 | 0 | 0 | 4 | 0 | 0.02% | 99.2% |
| 94 | 35 | Lascolabacillus massiliensis strain SIT8 (NR 144720.1) | 29 | 0 | 2 | 0 | 4 | 0 | 0.02% | 99.2% |
| 91 | 35 | Pedobacter sp. MIC2002 (JX978785) | 13 | 0 | 18 | 0 | 4 | 0 | 0.02% | 99.3% |
| 95 | 33 | Alkalibacter saccharofermentans (T) Z-79820 (AY312403) | 18 | 0 | 9 | 0 | 6 | 0 | 0.02% | 99.3% |
| 92 | 33 | Pedomicrobium manganicum (T) ATCC 33121 (GU269549) | 20 | 11 | 1 | 0 | 1 | 0 | 0.02% | 99.3% |
| 90 | 33 | Thermincola carboxydiphila (T) 2204 (AY603000) | 9 | 0 | 10 | 0 | 14 | 0 | 0.02% | 99.3% |
| 110 | 31 | Alkalitalea saponilacus (T) SC/BZ-SP2 (HQ191474) | 30 | 0 | 1 | 0 | 0 | 0 | 0.02% | 99.3% |
| 88 | 28 | Syntrophomonas zehnderi (T) OL-4 (DQ898277) | 0 | 0 | 2 | 0 | 2 | 24 | 0.02% | 99.4% |
| 105 | 27 | Bacillaceae bacterium 13CC (JN571119) | 10 | 0 | 10 | 0 | 7 | 0 | 0.01% | 99.4% |
| 121 | 27 | Pseudomonas aestusnigri CCUG 64165; VGXO14 (HG004394) | 18 | 0 | 4 | 0 | 5 | 0 | 0.01% | 99.4% |
| 104 | 25 | Vallitalea pronyensis FatNI3 (KC876639) | 1 | 0 | 0 | 0 | 17 | 7 | 0.01% | 99.4% |
| 106 | 25 | Lutaonella thermophila (T) CC-MHSW-2 (EU287913) | 15 | 0 | 5 | 0 | 5 | 0 | 0.01% | 99.4% |
| 132 | 25 | Olegusella massiliensis strain KHD7 (NR 146815.1) | 18 | 0 | 2 | 0 | 5 | 0 | 0.01% | 99.4% |
| 89 | 25 | Methanolinea mesophila TNR (AB447467) | 0 | 0 | 0 | 0 | 6 | 19 | 0.01% | 99.4% |
| 119 | 25 | Clostridium acetireducens (T) 30A (X79862) | 25 | 0 | 0 | 0 | 0 | 0 | 0.01% | 99.4% |
| 146 | 24 | Anaerobacterium chartisolvens T-1-35 (AB793710) | 11 | 0 | 9 | 0 | 4 | 0 | 0.01% | 99.5% |
| 101 | 24 | Methanobrevibacter boviskoreani JH1 (KC608769) | 23 | 0 | 1 | 0 | 0 | 0 | 0.01% | 99.5% |
| 107 | 24 | Ornatilinea apprima P3M-1 (JQ292916) | 0 | 0 | 2 | 0 | 8 | 14 | 0.01% | 99.5% |
| 113 | 23 | Spiribacter salinus M19-40 (CP005963) | 14 | 0 | 5 | 0 | 4 | 0 | 0.01% | 99.5% |
| 103 | 23 | Pelotomaculum thermopropionicum (T) SI (AB035723) | 0 | 0 | 0 | 0 | 16 | 7 | 0.01% | 99.5% |
| 108 | 22 | Gelria glutamica (T) TGO (AF321086) | 8 | 0 | 4 | 0 | 10 | 0 | 0.01% | 99.5% |
| 126 | 21 | Pelospora glutarica (T) WoGl3 (AJ251214) | 6 | 0 | 1 | 0 | 11 | 3 | 0.01% | 99.5% |
| 118 | 21 | Syntrophomonas zehnderi (T) OL-4 (DQ898277) | 11 | 0 | 9 | 0 | 1 | 0 | 0.01% | 99.5% |
| 112 | 20 | Lactobacillus gasseri (T) ATCC 33323 (AF519171) | 0 | 0 | 13 | 0 | 7 | 0 | 0.01% | 99.6% |
| 97 | 20 | Sphaerochaeta globus str. Buddy (AF357916) | 0 | 20 | 0 | 0 | 0 | 0 | 0.01% | 99.6% |
| 120 | 19 | Streptomyces aomiensis (T) M24DS04 (AB522686) | 11 | 0 | 0 | 0 | 8 | 0 | 0.01% | 99.6% |
| 109 | 19 | Desulfitibacter alkalitolerans (T) sk.kt5 (AY538171) | 0 | 0 | 1 | 0 | 12 | 6 | 0.01% | 99.6% |
| 117 | 18 | Mariniphaga sediminis strain SY21 (NR 137221.1) | 16 | 0 | 1 | 0 | 1 | 0 | 0.01% | 99.6% |
| 208 | 18 | Olegusella massiliensis strain KHD7 (NR 146815.1) | 11 | 0 | 4 | 0 | 3 | 0 | 0.01% | 99.6% |
| 145 | 17 | Cytophaga xylanolytica strain DSM 6779 (NR 117112.1) | 5 | 0 | 6 | 0 | 6 | 0 | 0.01% | 99.6% |
| 134 | 16 | Parvibaculum lavamentivorans (T) DS-1 (AY387398) | 5 | 0 | 7 | 0 | 4 | 0 | 0.01% | 99.6% |
| 123 | 16 | Natranaerovirga pectinivora (T) AP3 (GQ922846) | 5 | 0 | 6 | 0 | 5 | 0 | 0.01% | 99.6% |
| 130 | 15 | Melioribacter roseus P3M-2 (JQ292917) | 6 | 0 | 5 | 0 | 4 | 0 | 0.01% | 99.6% |
| 144 | 15 | Sunxiuqinia faeciviva (T) JAM-BA0302 (AB362263) | 5 | 0 | 6 | 0 | 4 | 0 | 0.01% | 99.7% |
| 155 | 14 | Acholeplasma parvum (T) H23M (AY538170) | 6 | 0 | 3 | 0 | 5 | 0 | 0.01% | 99.7% |
| 115 | 14 | Akkermansia muciniphila (T) Muc (AY271254) | 14 | 0 | 0 | 0 | 0 | 0 | 0.01% | 99.7% |
| 111 | 14 | Muribaculum intestinale strain YL27 (NR 144616.1) | 5 | 0 | 6 | 0 | 3 | 0 | 0.01% | 99.7% |
| 131 | 13 | Gracilibacter thermotolerans (T) JW/YJL-S1 (DQ117465) | 1 | 0 | 3 | 0 | 9 | 0 | 0.01% | 99.7% |
| 140 | 13 | [Clostridium] caenicola strain EBR596 (NR 126170.1) | 1 | 0 | 0 | 0 | 11 | 1 | 0.01% | 99.7% |
| 149 | 13 | Thermovirga lienii (T) Cas60314 (DQ071273) | 1 | 0 | 7 | 0 | 5 | 0 | 0.01% | 99.7% |
| 171 | 12 | Methanobacterium oryzae (T) FPi (AF028690) | 2 | 0 | 8 | 0 | 2 | 0 | 0.01% | 99.7% |
| 114 | 12 | Vallitalea pronyensis FatNI3 (KC876639) | 0 | 0 | 0 | 0 | 0 | 12 | 0.01% | 99.7% |
| 124 | 12 | Lactobacillus faecis AFL13-2 (AB812750) | 0 | 0 | 7 | 0 | 5 | 0 | 0.01% | 99.7% |
| 127 | 12 | Desulfuribacillus alkaliarsenatis AHT28 (HM046584) | 1 | 0 | 1 | 0 | 10 | 0 | 0.01% | 99.7% |
| 139 | 12 | Paracoccus aestuarii (T) B7 (EF660757) | 5 | 0 | 4 | 0 | 3 | 0 | 0.01% | 99.7% |
| 116 | 12 | Muribaculum intestinale strain YL27 (NR 144616.1) | 5 | 0 | 4 | 0 | 3 | 0 | 0.01% | 99.7% |
| 125 | 11 | Desulfosporosinus orientis (T) DSM 765 (Y11570) | 0 | 0 | 0 | 0 | 11 | 0 | 0.01% | 99.7% |
| 142 | 11 | Smithella propionica (T) LYP (AF126282) | 9 | 0 | 1 | 0 | 1 | 0 | 0.01% | 99.8% |
| 122 | 11 | Culturomica massiliensis strain Marseille-P2698 (NR 144745.1) | 0 | 0 | 9 | 0 | 2 | 0 | 0.01% | 99.8% |
| 128 | 10 | Porphyromonas pogonae strain MI 10-1288 (NR 136443.1) | 5 | 0 | 0 | 0 | 5 | 0 | 0.01% | 99.8% |
| 133 | 10 | Bellilinea caldifistulae (T) GOMI-1 (AB243672) | 0 | 0 | 4 | 0 | 6 | 0 | 0.01% | 99.8% |
| 148 | 10 | Acholeplasma parvum (T) H23M (AY538170) | 1 | 0 | 2 | 0 | 7 | 0 | 0.01% | 99.8% |
| 211 | 10 | Natronoflexus pectinivorans AP1 (GQ922844) | 3 | 0 | 2 | 0 | 5 | 0 | 0.01% | 99.8% |
| 135 | 10 | Soehngenia saccharolytica (T) BOR-Y (AY353956) | 4 | 0 | 2 | 0 | 4 | 0 | 0.01% | 99.8% |
| 186 | 10 | Caloramator fervidus (T) RT4. B1 (L09187) | 0 | 0 | 9 | 0 | 1 | 0 | 0.01% | 99.8% |
| 184 | 10 | Desulfitobacterium hafniense (T) DCB-2 (CP001336) | 3 | 0 | 0 | 0 | 7 | 0 | 0.01% | 99.8% |
| 154 | 9 | Geovibrio ferrireducens (T) PAL-1 (X95744) | 3 | 0 | 5 | 0 | 1 | 0 | 0.00% | 99.8% |
| 143 | 9 | Treponema caldarium (T) DSMZ7334 (EU580141) | 8 | 0 | 0 | 0 | 1 | 0 | 0.00% | 99.8% |
| 147 | 9 | Anoxynatronum sibiricum (T) Z-7981 (AF522323) | 7 | 0 | 1 | 0 | 1 | 0 | 0.00% | 99.8% |
| 157 | 9 | Thermanaerovibrio acidaminovorans strain DSM 6589 (NR 114455.1) | 0 | 0 | 9 | 0 | 0 | 0 | 0.00% | 99.8% |
| 152 | 9 | Moorella glycerini (T) YS6 (U82327) | 0 | 0 | 3 | 0 | 6 | 0 | 0.00% | 99.8% |
| 177 | 9 | Muribaculum intestinale strain YL27 (NR 144616.1) | 2 | 0 | 2 | 0 | 5 | 0 | 0.00% | 99.8% |
| 136 | 9 | Gracilibacter thermotolerans (T) JW/YJL-S1 (DQ117465) | 0 | 0 | 0 | 0 | 0 | 9 | 0.00% | 99.8% |
| 172 | 9 | Cytophaga fermentans (T) ATCC 19072 (M58766) | 0 | 0 | 5 | 0 | 4 | 0 | 0.00% | 99.8% |
| 178 | 9 | Desulfovibrio psychrotolerans (T) type strain: PWC = JS1 (AM418397) | 7 | 0 | 1 | 0 | 1 | 0 | 0.00% | 99.8% |
| 164 | 9 | Unclassified Bacteria | 3 | 0 | 4 | 0 | 2 | 0 | 0.00% | 99.8% |
| 137 | 8 | Christensenella minuta YIT 12065 (AB490809) | 8 | 0 | 0 | 0 | 0 | 0 | 0.00% | 99.8% |
| 150 | 8 | Petrimonas sulfuriphila (T) BN3 (AY570690) | 7 | 0 | 0 | 0 | 1 | 0 | 0.00% | 99.9% |
| 129 | 8 | Unclassified Firmicutes | 3 | 0 | 3 | 0 | 2 | 0 | 0.00% | 99.9% |
| 163 | 8 | Muribaculum intestinale strain YL27 (NR 144616.1) | 2 | 0 | 3 | 0 | 3 | 0 | 0.00% | 99.9% |
| 156 | 7 | Aminobacterium colombiense strain DSM 12261 (NR 074624.1) | 4 | 0 | 2 | 0 | 1 | 0 | 0.00% | 99.9% |
| 224 | 7 | Ornatilinea apprima P3M-1 (JQ292916) | 3 | 0 | 3 | 0 | 1 | 0 | 0.00% | 99.9% |
| 223 | 7 | Alkaliphilus peptidifermentans (T) Z-7036 (EF382660) | 4 | 0 | 1 | 0 | 2 | 0 | 0.00% | 99.9% |
| 141 | 7 | anaerobic bacterium MO-CFX1 (AB598277) | 0 | 0 | 0 | 0 | 7 | 0 | 0.00% | 99.9% |
| 153 | 7 | Unclassified Proteobacteria | 1 | 0 | 5 | 0 | 1 | 0 | 0.00% | 99.9% |
| 175 | 7 | Smithella propionica (T) LYP (AF126282) | 2 | 0 | 1 | 0 | 4 | 0 | 0.00% | 99.9% |
| 138 | 7 | Methanobacterium subterraneum (T) A8p, DSM 11074 (X99044) | 0 | 0 | 0 | 0 | 0 | 7 | 0.00% | 99.9% |
| 174 | 7 | Acetanaerobacterium elongatum (T) Z7 (AY487928) | 2 | 0 | 1 | 0 | 4 | 0 | 0.00% | 99.9% |
| 203 | 7 | Dethiobacter alkaliphilus (T) AHT 1 (EF422412) | 0 | 0 | 3 | 0 | 4 | 0 | 0.00% | 99.9% |
| 151 | 7 | Muribaculum intestinale strain YL27 (NR 144616.1) | 0 | 0 | 5 | 0 | 2 | 0 | 0.00% | 99.9% |
| 170 | 6 | Desulfuromonas thiophila (T) NZ27 (DSMZ 8987) (Y11560) | 5 | 0 | 1 | 0 | 0 | 0 | 0.00% | 99.9% |
| 162 | 6 | Thermanaerothrix daxensis strain GNS-1 (NR 117865.1) | 2 | 0 | 4 | 0 | 0 | 0 | 0.00% | 99.9% |
| 180 | 6 | Sedimentibacter saalensis (T) ZF2 (AJ404680) | 1 | 0 | 3 | 0 | 2 | 0 | 0.00% | 99.9% |
| 216 | 6 | Thermotogales bacterium MesG1Ag4.2.16S.B (HM003109) | 3 | 0 | 3 | 0 | 0 | 0 | 0.00% | 99.9% |
| 161 | 6 | Calditerricola yamamurae (T) YMO722 (AB308475) | 3 | 0 | 1 | 0 | 2 | 0 | 0.00% | 99.9% |
| 168 | 6 | Citrobacter sedlakii (T) CDC 4696-86 (AF025364) | 4 | 0 | 1 | 0 | 1 | 0 | 0.00% | 99.9% |
| 158 | 6 | Thermoanaerobacter thermocopriae (T) JT-3T (L09167) | 6 | 0 | 0 | 0 | 0 | 0 | 0.00% | 99.9% |
| 194 | 6 | Smithella propionica (T) LYP (AF126282) | 5 | 0 | 1 | 0 | 0 | 0 | 0.00% | 99.9% |
| 159 | 6 | Alkaliphilus crotonatoxidans (T) B11-2 (AF467248) | 5 | 0 | 1 | 0 | 0 | 0 | 0.00% | 99.9% |
| 191 | 5 | Moorella humiferrea (T) 64 FGQ (GQ872425) | 4 | 0 | 0 | 0 | 1 | 0 | 0.00% | 99.9% |
| 198 | 5 | Methanofollis tationis (T) DSM 2702 (AF095272) | 3 | 0 | 1 | 0 | 1 | 0 | 0.00% | 99.9% |
| 187 | 5 | Streptomyces cinereorectus (T) NBRC 15395 (AB184646) | 0 | 0 | 5 | 0 | 0 | 0 | 0.00% | 99.9% |
| 200 | 5 | Levilinea saccharolytica (T) KIBI-1 (AB109439) | 0 | 0 | 4 | 0 | 1 | 0 | 0.00% | 99.9% |
| 167 | 5 | Anaerobranca zavarzinii (T) JW/VK-KS5Y (EF190921) | 1 | 0 | 3 | 0 | 1 | 0 | 0.00% | 99.9% |
| 169 | 5 | Defluviitalea saccharophila (T) LIND6LT2 (HQ020487) | 1 | 0 | 0 | 0 | 4 | 0 | 0.00% | 99.9% |
| 182 | 5 | Muribaculum intestinale strain YL27 (NR 144616.1) | 2 | 0 | 1 | 0 | 2 | 0 | 0.00% | 99.9% |
| 217 | 4 | Lutispora thermophila (T) EBR46 (AB186360) | 3 | 0 | 1 | 0 | 0 | 0 | 0.00% | 100.0% |
| 221 | 4 | Cytophaga xylanolytica strain DSM 6779 (NR 117112.1) | 1 | 0 | 2 | 0 | 1 | 0 | 0.00% | 100.0% |
| 219 | 4 | uncultured candidate division BRC1 bacterium LD1-PA21 (AY114315) | 4 | 0 | 0 | 0 | 0 | 0 | 0.00% | 100.0% |
| 160 | 4 | Mesorhizobium camelthorni (T) CCNWXJ40-4 (EU169581) | 2 | 0 | 2 | 0 | 0 | 0 | 0.00% | 100.0% |
| 185 | 4 | Muribaculum intestinale strain YL27 (NR 144616.1) | 0 | 0 | 1 | 0 | 3 | 0 | 0.00% | 100.0% |
| 193 | 4 | Gelria glutamica (T) TGO (AF321086) | 0 | 0 | 4 | 0 | 0 | 0 | 0.00% | 100.0% |
| 173 | 4 | Smithella propionica (T) LYP (AF126282) | 2 | 0 | 2 | 0 | 0 | 0 | 0.00% | 100.0% |
| 176 | 4 | Pleomorphomonas diazotrophica R5-392 (JQ346801) | 1 | 0 | 3 | 0 | 0 | 0 | 0.00% | 100.0% |
| 181 | 4 | Desulfotomaculum acetoxidans (T) DSM 771 (Y11566) | 1 | 0 | 2 | 0 | 1 | 0 | 0.00% | 100.0% |
| 179 | 4 | Pseudobacteroides cellulosolvens (L35517) | 0 | 0 | 0 | 0 | 4 | 0 | 0.00% | 100.0% |
| 183 | 3 | Muribaculum intestinale strain YL27 (NR 144616.1) | 2 | 0 | 0 | 0 | 1 | 0 | 0.00% | 100.0% |
| 202 | 3 | Muribaculum intestinale strain YL27 (NR 144616.1) | 0 | 0 | 1 | 0 | 2 | 0 | 0.00% | 100.0% |
| 188 | 3 | Gracilibacter thermotolerans (T) JW/YJL-S1 (DQ117465) | 0 | 0 | 2 | 0 | 1 | 0 | 0.00% | 100.0% |
| 192 | 3 | Unclassified ;naerolineaceae | 3 | 0 | 0 | 0 | 0 | 0 | 0.00% | 100.0% |
| 207 | 3 | Candidatus Procabacter acanthamoebae (AF177427) | 2 | 0 | 1 | 0 | 0 | 0 | 0.00% | 100.0% |
| 197 | 3 | Muribaculum intestinale strain YL27 (NR 144616.1) | 1 | 0 | 0 | 0 | 2 | 0 | 0.00% | 100.0% |
| 213 | 3 | uncultured bacterium FW34 (AF523981) | 3 | 0 | 0 | 0 | 0 | 0 | 0.00% | 100.0% |
| 218 | 3 | uncultured bacterium FW34 (AF523981) | 3 | 0 | 0 | 0 | 0 | 0 | 0.00% | 100.0% |
| 212 | 3 | Methanoregula formicica (T) SMSP (AB479390) | 3 | 0 | 0 | 0 | 0 | 0 | 0.00% | 100.0% |
| 210 | 3 | Clostridium saccharolyticum (T) DSM 2544 (Y18185) | 0 | 0 | 0 | 0 | 3 | 0 | 0.00% | 100.0% |
| 190 | 2 | Acinetobacter indicus (T) A648 (HM047743) | 0 | 0 | 0 | 0 | 2 | 0 | 0.00% | 100.0% |
| 209 | 2 | Dehalobacter restrictus (T) PER-K23 (U84497) | 2 | 0 | 0 | 0 | 0 | 0 | 0.00% | 100.0% |
| 220 | 2 | Bacteroides acidifaciens (T) A40 (AB021164) | 1 | 0 | 1 | 0 | 0 | 0 | 0.00% | 100.0% |
| 215 | 2 | Saccharofermentans acetigenes (T) P6 (AY949857) | 2 | 0 | 0 | 0 | 0 | 0 | 0.00% | 100.0% |
| 189 | 2 | Ornatilinea apprima P3M-1 (JQ292916) | 0 | 0 | 1 | 0 | 1 | 0 | 0.00% | 100.0% |
| 222 | 2 | Ruminococcaceae bacterium ZWB 4 (HG003571) | 0 | 0 | 1 | 0 | 1 | 0 | 0.00% | 100.0% |
| 201 | 2 | Clostridium lavalense (T) CCRI-9842 (EF564277) | 2 | 0 | 0 | 0 | 0 | 0 | 0.00% | 100.0% |
| 195 | 2 | Desulfurispora thermophila (T) RA50E1 (AY548776) | 0 | 0 | 2 | 0 | 0 | 0 | 0.00% | 100.0% |
| 214 | 2 | Candidatus Cloacimonas acidaminovorans str. Evry (CU466930) | 2 | 0 | 0 | 0 | 0 | 0 | 0.00% | 100.0% |
| 205 | 2 | Hydrogenophaga defluvii (T) type strain: BSB 9.5 (AJ585993) | 0 | 0 | 0 | 0 | 2 | 0 | 0.00% | 100.0% |
| 206 | 2 | uncultured soil bacterium PBS-III-27 (AJ390456) | 0 | 0 | 2 | 0 | 0 | 0 | 0.00% | 100.0% |
