# Supplementary material for: Kelpie: generating full-length ‘amplicons’ from whole-metagenome datasets
Source: PeerJ. 2019 Jan 30;6:e6174. doi: 10.7717/peerj.6174 (PMC6359901; doi:10.7717/peerj.6174)
Supplement: Table S3 — Full OTU table produced from amplicon, Kelpie-generated sequences and primer-bounded sequences extracted from full metaSPAdes assemblies for each WGS sample file, and from assemblies of the filtered 16S region reads, for all three coal seam microbiome samples, ordered by total abundance. Red amplicon counts indicated that mapping the WGS reads for the sample back to the consensus sequence for the OTU showed that it had less than 90% kMer coverage. The green amplicon counts show where the WGS reads for a sample gave 100% coverage of the OTU sequence. This table is derived from the ‘AESS’ tabs in Table S6. [file peerj-07-6174-s003.docx]

Table S3: Full OTU table produced from **amplicon, Kelpie-generated sequences and primer-bounded sequences extracted from full metaSPAdes assemblies for each sample, and from assemblies of the filtered 16S region reads**, for all three coal seam microbiome samples, ordered by total abundance. Red amplicon counts indicated that mapping the WGS reads for the sample back to the consensus sequence for the OTU showed that it had less than 90% kMer coverage. The green amplicon counts show where the WGS reads for a sample gave 100% coverage of the OTU sequence. The orange fill indicates cells where organisms were detected using the amplicons and Kelpie but were missed by one or other of the metaSPAdes assemblies. This table is derived from the ‘AESS’ tabs in the Excel spreadsheet ‘Kelpie - CSM.xlsx’ which is available as Supplemental Table S6.

| OTU | Size | CSM Species | W1 amp. | W1 16S ext. | W1 Spades contigs | W1 Spades 16S contigs | W2 amp. | W2 16S ext. | W2 Spades contigs | W2 Spades 16S contigs | W3 amp. | W3 16S ext. | W3 Spades contigs | W3 Spades 16S contigs |
| --- | --- | --- | --- | --- | --- | --- | --- | --- | --- | --- | --- | --- | --- | --- |
| 1 | 58184 | Desulfuromonas acetexigens (T) (U23140) | 27333 | 13574 | 5292 | 8544 | 132 | 0 | 0 | 0 | 1554 | 1010 | 195 | 550 |
| 2 | 24970 | Thermodesulfovibrio aggregans (T) TGE-P1 (AB021302) | 24 | 0 | 0 | 0 | 17120 | 7816 | 0 | 0 | 10 | 0 | 0 | 0 |
| 3 | 14236 | Treponema zuelzerae (T) type strain: DSM 1903; 2 (FR749929) | 13 | 0 | 0 | 0 | 1171 | 305 | 122 | 229 | 5956 | 3069 | 1889 | 1482 |
| 4 | 12568 | Methanobacterium subterraneum (T) A8p, DSM 11074 (X99044) | 5 | 0 | 0 | 0 | 29 | 0 | 0 | 0 | 7736 | 2393 | 1396 | 1009 |
| 6 | 8872 | Parabacteroides distasonis (T) JCM 5825 (AB238922) | 1270 | 271 | 193 | 140 | 9 | 0 | 0 | 0 | 3116 | 1598 | 1207 | 1068 |
| 7 | 8470 | Methanosaeta harundinacea (T) 8Ac (AY817738) | 1032 | 192 | 0 | 0 | 16 | 0 | 0 | 0 | 3332 | 1942 | 1044 | 912 |
| 8 | 8389 | Cytophaga fermentans (T) ATCC 19072 (M58766) | 9 | 0 | 0 | 0 | 5845 | 1220 | 766 | 542 | 7 | 0 | 0 | 0 |
| 5 | 8105 | Methanosarcina siciliae type strain: DSM3028 (FR733698) | 1238 | 2733 | 1738 | 2109 | 11 | 0 | 0 | 0 | 54 | 222 | 0 | 0 |
| 11 | 5998 | Lysinibacillus sp. LAM612 (KF443809) | 3 | 0 | 0 | 0 | 7 | 0 | 0 | 0 | 533 | 4068 | 0 | 1387 |
| 10 | 5619 | candidate division OP1 clone OPB14 (AF027045) | 3 | 0 | 0 | 0 | 4057 | 771 | 424 | 358 | 6 | 0 | 0 | 0 |
| 9 | 5520 | Thermacetogenium phaeum (T) PB (AB020336) | 5 | 0 | 0 | 0 | 14 | 0 | 0 | 0 | 3285 | 2216 | 0 | 0 |
| 12 | 4395 | Sulfurospirillum alkalitolerans HTRB-L1 (GQ863490) | 2340 | 508 | 0 | 1475 | 41 | 0 | 0 | 0 | 31 | 0 | 0 | 0 |
| 13 | 4223 | Syntrophaceticus schinkii (T) Sp3 (EU386162) | 6 | 0 | 0 | 0 | 2871 | 506 | 400 | 433 | 7 | 0 | 0 | 0 |
| 14 | 4165 | Methanocalculus pumilus (T) MHT-1 (AB008853) | 3312 | 476 | 0 | 318 | 30 | 0 | 0 | 0 | 29 | 0 | 0 | 0 |
| 16 | 3899 | Methanobacterium aarhusense (T) H2-LR (AY386124) | 1 | 0 | 0 | 0 | 3104 | 271 | 253 | 263 | 7 | 0 | 0 | 0 |
| 15 | 3652 | Desulfotomaculum acetoxidans (T) DSM 771 (Y11566) | 6 | 0 | 0 | 0 | 2463 | 1177 | 0 | 0 | 6 | 0 | 0 | 0 |
| 19 | 3012 | Methanothermobacter thermoflexus (T) IDZ, VKM B-1963, DSM 7268 (X99047) | 1 | 0 | 0 | 0 | 2685 | 326 | 0 | 0 | 0 | 0 | 0 | 0 |
| 24 | 2540 | Natronincola peptidivorans (T) Z-7031 (EF382661) | 12 | 0 | 0 | 0 | 8 | 0 | 0 | 0 | 1293 | 754 | 0 | 473 |
| 18 | 2287 | * | 2 | 0 | 0 | 0 | 955 | 556 | 482 | 292 | 0 | 0 | 0 | 0 |
| 17 | 2245 | Caldicoprobacter guelmensis (T) D2C22 (JQ707908) | 17 | 0 | 0 | 0 | 7 | 0 | 0 | 0 | 477 | 343 | 946 | 455 |
| 29 | 2114 | Methanobacterium alcaliphilum (T) NBRC 105226 (AB496639) | 2 | 0 | 0 | 0 | 1161 | 100 | 0 | 0 | 586 | 265 | 0 | 0 |
| 20 | 2099 | Clostridium hungatei (T) AD; ATCC 700212 (AF020429) | 5 | 0 | 0 | 0 | 5 | 0 | 0 | 0 | 1124 | 965 | 0 | 0 |
| 23 | 2005 | Ignavibacterium album (T) Mat9-16 (AB478415) | 1 | 0 | 0 | 0 | 1089 | 408 | 236 | 262 | 9 | 0 | 0 | 0 |
| 27 | 1974 | Porphyromonas pogonae strain MI 10-1288 (NR 136443.1) | 1059 | 128 | 45 | 61 | 29 | 0 | 0 | 0 | 389 | 129 | 71 | 63 |
| 21 | 1955 | Pontibacter sp. JC215 A10 (HG008901) | 4 | 0 | 0 | 0 | 2 | 0 | 0 | 0 | 931 | 1018 | 0 | 0 |
| 22 | 1941 | Desulfovibrio oxamicus (T) DSM 1925 (DQ122124) | 19 | 10 | 0 | 0 | 2 | 0 | 0 | 0 | 860 | 624 | 426 | 0 |
| 26 | 1857 | Sporomusa ovata strain DSM 2662 (NR 117659.1) | 1 | 0 | 0 | 0 | 0 | 0 | 0 | 0 | 306 | 175 | 805 | 570 |
| 28 | 1822 | Acetobacterium malicum (T) DSM 4132 (X96957) | 929 | 304 | 37 | 161 | 16 | 0 | 0 | 0 | 153 | 155 | 67 | 0 |
| 25 | 1536 | Moorella humiferrea (T) 64 FGQ (GQ872425) | 1 | 0 | 0 | 0 | 129 | 847 | 559 | 0 | 0 | 0 | 0 | 0 |
| 31 | 1351 | Thiohalocapsa marina (T) type strain: JA142 (AM491592) | 304 | 98 | 50 | 894 | 0 | 0 | 0 | 0 | 5 | 0 | 0 | 0 |
| 41 | 1322 | Ornatilinea apprima P3M-1 (JQ292916) | 796 | 254 | 154 | 113 | 3 | 0 | 0 | 0 | 2 | 0 | 0 | 0 |
| 30 | 1295 | Dethiobacter alkaliphilus (T) AHT 1 (EF422412) | 0 | 0 | 0 | 0 | 4 | 0 | 0 | 0 | 23 | 19 | 0 | 1249 |
| 33 | 1244 | Syntrophobacter sulfatireducens (T) TB8106 (AY651787) | 219 | 65 | 0 | 190 | 17 | 0 | 0 | 0 | 254 | 139 | 186 | 174 |
| 36 | 1224 | Thermodesulfovibrio aggregans (T) TGE-P1 (AB021302) | 2 | 0 | 0 | 0 | 722 | 440 | 59 | 0 | 1 | 0 | 0 | 0 |
| 32 | 1182 | Aminiphilus circumscriptus (T) ILE-2 (AY642589) | 0 | 0 | 0 | 0 | 0 | 0 | 0 | 0 | 0 | 4 | 1178 | 0 |
| 34 | 1063 | Clostridium sufflavum (T) CDT-1 (AB267266) | 13 | 14 | 0 | 997 | 0 | 0 | 0 | 0 | 27 | 12 | 0 | 0 |
| 35 | 1005 | Dielma fastidiosa strain JC13 (NR 125593.1) | 0 | 0 | 0 | 0 | 0 | 0 | 0 | 0 | 32 | 178 | 466 | 329 |
| 39 | 973 | Desulfovibrio alkalitolerans (T) RT2 (AY649785) | 108 | 23 | 8 | 129 | 4 | 0 | 0 | 0 | 429 | 23 | 135 | 114 |
| 38 | 944 | Magnetospira thiophila (T) MMS-1 (EU861390) | 6 | 0 | 0 | 8 | 5 | 0 | 0 | 0 | 290 | 285 | 202 | 148 |
| 37 | 910 | Desulfitibacter alkalitolerans (T) sk.kt5 (AY538171) | 0 | 0 | 0 | 0 | 1 | 0 | 0 | 0 | 13 | 6 | 241 | 649 |
| 40 | 866 | Pelotomaculum propionicicum (T) MGP (AB154390) | 4 | 0 | 0 | 0 | 188 | 67 | 43 | 564 | 0 | 0 | 0 | 0 |
| 49 | 774 | Dethiobacter alkaliphilus (T) AHT 1 (EF422412) | 4 | 0 | 0 | 0 | 43 | 84 | 18 | 147 | 116 | 99 | 16 | 247 |
| 42 | 749 | Sunxiuqinia faeciviva (T) JAM-BA0302 (AB362263) | 53 | 13 | 15 | 53 | 111 | 82 | 53 | 62 | 119 | 188 | 0 | 0 |
| 44 | 604 | Aminiphilus circumscriptus (T) ILE-2 (AY642589) | 205 | 82 | 53 | 259 | 2 | 0 | 0 | 0 | 3 | 0 | 0 | 0 |
| 43 | 601 | Aminivibrio pyruvatiphilus 4F6E (AB623229) | 128 | 62 | 6 | 403 | 2 | 0 | 0 | 0 | 0 | 0 | 0 | 0 |
| 45 | 563 | Smithella propionica (T) LYP (AF126282) | 7 | 0 | 0 | 0 | 217 | 65 | 0 | 0 | 82 | 25 | 77 | 90 |
| 50 | 472 | * | 0 | 0 | 0 | 0 | 259 | 211 | 0 | 0 | 2 | 0 | 0 | 0 |
| 172 | 461 | Pontibacter niistensis (T) NII-0905 (FJ897494) | 1 | 0 | 0 | 0 | 269 | 100 | 26 | 65 | 0 | 0 | 0 | 0 |
| 46 | 447 | Pseudomonas songnenensis strain NEAU-ST5-5 (NR 148295.1) | 6 | 0 | 0 | 0 | 1 | 0 | 0 | 0 | 138 | 126 | 102 | 74 |
| 65 | 441 | Smithella propionica (T) LYP (AF126282) | 84 | 33 | 0 | 269 | 3 | 0 | 0 | 0 | 27 | 25 | 0 | 0 |
| 48 | 425 | Syntrophomonas bryantii type strain: DSM 3014 (HE654006) | 53 | 21 | 0 | 12 | 4 | 0 | 0 | 0 | 61 | 69 | 0 | 205 |
| 47 | 409 | Methanospirillum hungatei strain JF-1 (NR 074177.1) | 67 | 135 | 0 | 206 | 0 | 0 | 0 | 0 | 1 | 0 | 0 | 0 |
| 51 | 397 | Dethiosulfatibacter aminovorans (T) C/G2 (= JCM 13356, = NBRC 101112, = DSM 17477) (AB218661) | 230 | 115 | 0 | 49 | 0 | 0 | 0 | 0 | 3 | 0 | 0 | 0 |
| 52 | 316 | uncultured bacterium KF-JG30-18 (AJ295656) | 0 | 0 | 0 | 0 | 44 | 28 | 23 | 221 | 0 | 0 | 0 | 0 |
| 67 | 315 | Bellilinea caldifistulae (T) GOMI-1 (AB243672) | 2 | 0 | 0 | 0 | 93 | 48 | 0 | 20 | 51 | 50 | 23 | 28 |
| 53 | 276 | Clostridium luticellarii strain FW431 (NR 145907.1) | 0 | 0 | 0 | 0 | 0 | 0 | 0 | 0 | 53 | 63 | 60 | 100 |
| 54 | 275 | Ruminococcaceae bacterium ZWB 4 (HG003571) | 169 | 47 | 31 | 25 | 2 | 0 | 0 | 0 | 1 | 0 | 0 | 0 |
| 55 | 265 | Desulfitobacterium metallireducens (T) 853-15A (AF297871) | 0 | 0 | 0 | 0 | 1 | 0 | 0 | 0 | 146 | 118 | 0 | 0 |
| 57 | 257 | Desulfitibacter alkalitolerans (T) sk.kt5 (AY538171) | 0 | 0 | 0 | 0 | 2 | 0 | 0 | 0 | 181 | 74 | 0 | 0 |
| 58 | 242 | Marivirga sericea (T) IFO 15983 (AB078081) | 125 | 58 | 9 | 41 | 8 | 0 | 0 | 0 | 1 | 0 | 0 | 0 |
| 59 | 232 | * | 161 | 70 | 0 | 0 | 1 | 0 | 0 | 0 | 0 | 0 | 0 | 0 |
| 56 | 230 | Desulfomicrobium salsuginis strain ADR21 (NR 132593.1) | 25 | 8 | 0 | 193 | 0 | 0 | 0 | 0 | 4 | 0 | 0 | 0 |
| 63 | 212 | Olivibacter sitiensis (T) AW-6 (DQ421387) | 175 | 33 | 0 | 0 | 0 | 0 | 0 | 0 | 4 | 0 | 0 | 0 |
| 60 | 201 | Petrimonas sulfuriphila (T) BN3 (AY570690) | 104 | 16 | 16 | 65 | 0 | 0 | 0 | 0 | 0 | 0 | 0 | 0 |
| 61 | 193 | Thermanaerothrix daxensis strain GNS-1 (NR 117865.1) | 0 | 0 | 0 | 0 | 70 | 79 | 0 | 44 | 0 | 0 | 0 | 0 |
| 195 | 192 | Caldicoprobacter algeriensis TH7C1 (GU216701) | 0 | 0 | 0 | 0 | 132 | 60 | 0 | 0 | 0 | 0 | 0 | 0 |
| 64 | 184 | Thermodesulfovibrio yellowstonii (T) YP87 (AB231858) | 0 | 0 | 0 | 0 | 107 | 77 | 0 | 0 | 0 | 0 | 0 | 0 |
| 62 | 178 | Spirochaeta smaragdinae (T) SEBR 4228; DSM 11293 (U80597) | 4 | 0 | 0 | 0 | 0 | 0 | 0 | 0 | 61 | 53 | 29 | 31 |
| 74 | 175 | Syntrophorhabdus aromaticivorans (T) UI (AB212873) | 25 | 3 | 0 | 0 | 47 | 19 | 0 | 0 | 25 | 56 | 0 | 0 |
| 69 | 157 | Clostridium thermocellum (T) ATCC 27405 (CP000568) | 0 | 0 | 0 | 0 | 2 | 0 | 0 | 0 | 106 | 49 | 0 | 0 |
| 70 | 148 | Ornatilinea apprima P3M-1 (JQ292916) | 109 | 34 | 0 | 0 | 2 | 0 | 0 | 0 | 3 | 0 | 0 | 0 |
| 68 | 134 | Tepidanaerobacter syntrophicus (T) JL (AB106353) | 0 | 0 | 0 | 0 | 0 | 0 | 0 | 0 | 32 | 40 | 62 | 0 |
| 66 | 129 | Paenibacillus phyllosphaerae (T) PALXIL04 (AY598818) | 0 | 0 | 0 | 129 | 0 | 0 | 0 | 0 | 0 | 0 | 0 | 0 |
| 76 | 127 | Azoarcus olearius DQS-4 (EF158388) | 71 | 18 | 15 | 0 | 10 | 0 | 0 | 0 | 13 | 0 | 0 | 0 |
| 72 | 125 | Syntrophorhabdus aromaticivorans (T) UI (AB212873) | 7 | 0 | 0 | 0 | 2 | 0 | 0 | 0 | 82 | 34 | 0 | 0 |
| 75 | 118 | Geoalkalibacter ferrihydriticus (T) Z-0531 (DQ309326) | 4 | 0 | 0 | 0 | 2 | 0 | 0 | 0 | 61 | 51 | 0 | 0 |
| 212 | 114 | Dethiobacter alkaliphilus (T) AHT 1 (EF422412) | 1 | 0 | 0 | 0 | 63 | 30 | 0 | 0 | 15 | 5 | 0 | 0 |
| 71 | 111 | Crocinitomix catalasitica (T) IFO 15977 (AB078042) | 38 | 37 | 6 | 28 | 2 | 0 | 0 | 0 | 0 | 0 | 0 | 0 |
| 78 | 101 | Dethiobacter alkaliphilus (T) AHT 1 (EF422412) | 0 | 0 | 0 | 0 | 58 | 41 | 0 | 0 | 2 | 0 | 0 | 0 |
| 73 | 92 | * | 0 | 0 | 0 | 0 | 0 | 0 | 0 | 0 | 11 | 40 | 23 | 18 |
| 79 | 92 | Smithella propionica (T) LYP (AF126282) | 59 | 32 | 0 | 0 | 1 | 0 | 0 | 0 | 0 | 0 | 0 | 0 |
| 80 | 86 | Desulfotomaculum kuznetsovii strain 17 (NR 115129.1) | 0 | 0 | 0 | 0 | 50 | 35 | 0 | 0 | 1 | 0 | 0 | 0 |
| 77 | 84 | Leptolinea tardivitalis (T) YMTK-2 (AB109438) | 0 | 0 | 0 | 0 | 29 | 37 | 18 | 0 | 0 | 0 | 0 | 0 |
| 85 | 83 | Smithella propionica (T) LYP (AF126282) | 54 | 24 | 0 | 0 | 3 | 0 | 0 | 0 | 2 | 0 | 0 | 0 |
| 82 | 79 | Atopobium vaginae (T) CCUG 38953 (Y17195) | 51 | 0 | 0 | 0 | 14 | 0 | 0 | 0 | 14 | 0 | 0 | 0 |
| 81 | 78 | * | 43 | 35 | 0 | 0 | 0 | 0 | 0 | 0 | 0 | 0 | 0 | 0 |
| 84 | 75 | Ruminococcaceae bacterium ZWB 4 (HG003571) | 4 | 0 | 0 | 0 | 1 | 0 | 0 | 0 | 46 | 24 | 0 | 0 |
| 83 | 71 | Longilinea arvoryzae (T) KOME-1 (AB243673) | 0 | 0 | 0 | 0 | 1 | 0 | 0 | 0 | 22 | 35 | 6 | 7 |
| 201 | 65 | Aminivibrio pyruvatiphilus 4F6E (AB623229) | 60 | 5 | 0 | 0 | 0 | 0 | 0 | 0 | 0 | 0 | 0 | 0 |
| 87 | 63 | Desulfotomaculum varum RH04-3 (GU126374) | 2 | 0 | 0 | 0 | 0 | 0 | 0 | 0 | 39 | 11 | 11 | 0 |
| 90 | 61 | Methanobacterium formicicum (T) DSM 1535 (AF169245) | 10 | 0 | 0 | 0 | 18 | 0 | 0 | 0 | 33 | 0 | 0 | 0 |
| 86 | 60 | Veillonella magna (T) lac18 (EU096495) | 5 | 0 | 0 | 0 | 0 | 0 | 0 | 0 | 28 | 27 | 0 | 0 |
| 91 | 53 | Sphingomonas ginsenosidimutans (T) Gsoil 1429 (HM204925) | 0 | 0 | 0 | 8 | 0 | 0 | 0 | 0 | 0 | 45 | 0 | 0 |
| 89 | 52 | Clostridium putrificum (T) DSM 1734 (X73442) | 23 | 23 | 0 | 0 | 4 | 0 | 0 | 0 | 2 | 0 | 0 | 0 |
| 88 | 52 | Acidobacteria bacterium P105 (KJ461654) | 0 | 0 | 0 | 0 | 31 | 21 | 0 | 0 | 0 | 0 | 0 | 0 |
| 100 | 47 | Pedomicrobium manganicum (T) ATCC 33121 (GU269549) | 20 | 11 | 0 | 14 | 1 | 0 | 0 | 0 | 1 | 0 | 0 | 0 |
| 103 | 45 | Gracilibacter thermotolerans (T) JW/YJL-S1 (DQ117465) | 18 | 0 | 0 | 0 | 0 | 0 | 0 | 0 | 14 | 13 | 0 | 0 |
| 106 | 44 | Proteiniphilum acetatigenes (T) TB107 (AY742226) | 27 | 6 | 0 | 0 | 4 | 0 | 0 | 0 | 7 | 0 | 0 | 0 |
| 98 | 42 | Olegusella massiliensis strain KHD7 (NR 146815.1) | 0 | 0 | 0 | 0 | 20 | 21 | 0 | 0 | 1 | 0 | 0 | 0 |
| 105 | 41 | Clostridium hungatei (T) AD; ATCC 700212 (AF020429) | 32 | 9 | 0 | 0 | 0 | 0 | 0 | 0 | 0 | 0 | 0 | 0 |
| 94 | 41 | Dethiobacter alkaliphilus (T) AHT 1 (EF422412) | 15 | 0 | 0 | 0 | 14 | 0 | 0 | 0 | 12 | 0 | 0 | 0 |
| 92 | 38 | Clostridium thermopalmarium (T) (X72869) | 0 | 0 | 0 | 0 | 0 | 0 | 0 | 0 | 17 | 21 | 0 | 0 |
| 93 | 36 | Methanolinea mesophila TNR (AB447467) | 0 | 0 | 0 | 0 | 0 | 0 | 0 | 0 | 6 | 19 | 11 | 0 |
| 102 | 35 | Lascolabacillus massiliensis strain SIT8 (NR 144720.1) | 29 | 0 | 0 | 0 | 2 | 0 | 0 | 0 | 4 | 0 | 0 | 0 |
| 99 | 35 | Pedobacter sp. MIC2002 (JX978785) | 13 | 0 | 0 | 0 | 18 | 0 | 0 | 0 | 4 | 0 | 0 | 0 |
| 97 | 33 | Thermincola carboxydiphila (T) 2204 (AY603000) | 9 | 0 | 0 | 0 | 10 | 0 | 0 | 0 | 14 | 0 | 0 | 0 |
| 101 | 33 | Alkalibacter saccharofermentans (T) Z-79820 (AY312403) | 18 | 0 | 0 | 0 | 9 | 0 | 0 | 0 | 6 | 0 | 0 | 0 |
| 113 | 31 | Alkalitalea saponilacus (T) SC/BZ-SP2 (HQ191474) | 30 | 0 | 0 | 0 | 1 | 0 | 0 | 0 | 0 | 0 | 0 | 0 |
| 95 | 28 | Sphaerochaeta globus str. Buddy (AF357916) | 0 | 20 | 8 | 0 | 0 | 0 | 0 | 0 | 0 | 0 | 0 | 0 |
| 96 | 28 | Syntrophomonas zehnderi (T) OL-4 (DQ898277) | 0 | 0 | 0 | 0 | 2 | 0 | 0 | 0 | 2 | 24 | 0 | 0 |
| 104 | 28 | Methanobrevibacter boviskoreani JH1 (KC608769) | 23 | 0 | 4 | 0 | 1 | 0 | 0 | 0 | 0 | 0 | 0 | 0 |
| 108 | 27 | Bacillaceae bacterium 13CC (JN571119) | 10 | 0 | 0 | 0 | 10 | 0 | 0 | 0 | 7 | 0 | 0 | 0 |
| 126 | 27 | Pseudomonas aestusnigri CCUG 64165; VGXO14 (HG004394) | 18 | 0 | 0 | 0 | 4 | 0 | 0 | 0 | 5 | 0 | 0 | 0 |
| 107 | 25 | Vallitalea pronyensis FatNI3 (KC876639) | 1 | 0 | 0 | 0 | 0 | 0 | 0 | 0 | 17 | 7 | 0 | 0 |
| 112 | 25 | Lutaonella thermophila (T) CC-MHSW-2 (EU287913) | 15 | 0 | 0 | 0 | 5 | 0 | 0 | 0 | 5 | 0 | 0 | 0 |
| 124 | 25 | Clostridium acetireducens (T) 30A (X79862) | 25 | 0 | 0 | 0 | 0 | 0 | 0 | 0 | 0 | 0 | 0 | 0 |
| 147 | 24 | Anaerobacterium chartisolvens T-1-35 (AB793710) | 11 | 0 | 0 | 0 | 9 | 0 | 0 | 0 | 4 | 0 | 0 | 0 |
| 110 | 24 | Ornatilinea apprima P3M-1 (JQ292916) | 0 | 0 | 0 | 0 | 2 | 0 | 0 | 0 | 8 | 14 | 0 | 0 |
| 117 | 23 | Spiribacter salinus M19-40 (CP005963) | 14 | 0 | 0 | 0 | 5 | 0 | 0 | 0 | 4 | 0 | 0 | 0 |
| 109 | 23 | Pelotomaculum thermopropionicum (T) SI (AB035723) | 0 | 0 | 0 | 0 | 0 | 0 | 0 | 0 | 16 | 7 | 0 | 0 |
| 111 | 22 | Gelria glutamica (T) TGO (AF321086) | 8 | 0 | 0 | 0 | 4 | 0 | 0 | 0 | 10 | 0 | 0 | 0 |
| 131 | 21 | Pelospora glutarica (T) WoGl3 (AJ251214) | 6 | 0 | 0 | 0 | 1 | 0 | 0 | 0 | 11 | 3 | 0 | 0 |
| 121 | 21 | Syntrophomonas zehnderi (T) OL-4 (DQ898277) | 11 | 0 | 0 | 0 | 9 | 0 | 0 | 0 | 1 | 0 | 0 | 0 |
| 114 | 20 | Lactobacillus gasseri (T) ATCC 33323 (AF519171) | 0 | 0 | 0 | 0 | 13 | 0 | 0 | 0 | 7 | 0 | 0 | 0 |
| 122 | 19 | Streptomyces aomiensis (T) M24DS04 (AB522686) | 11 | 0 | 0 | 0 | 0 | 0 | 0 | 0 | 8 | 0 | 0 | 0 |
| 123 | 18 | Mariniphaga sediminis strain SY21 (NR 137221.1) | 16 | 0 | 0 | 0 | 1 | 0 | 0 | 0 | 1 | 0 | 0 | 0 |
| 148 | 17 | Cytophaga xylanolytica strain DSM 6779 (NR 117112.1) | 5 | 0 | 0 | 0 | 6 | 0 | 0 | 0 | 6 | 0 | 0 | 0 |
| 133 | 16 | Parvibaculum lavamentivorans (T) DS-1 (AY387398) | 5 | 0 | 0 | 0 | 7 | 0 | 0 | 0 | 4 | 0 | 0 | 0 |
| 129 | 16 | Natranaerovirga pectinivora (T) AP3 (GQ922846) | 5 | 0 | 0 | 0 | 6 | 0 | 0 | 0 | 5 | 0 | 0 | 0 |
| 132 | 15 | Melioribacter roseus P3M-2 (JQ292917) | 6 | 0 | 0 | 0 | 5 | 0 | 0 | 0 | 4 | 0 | 0 | 0 |
| 145 | 15 | Sunxiuqinia faeciviva (T) JAM-BA0302 (AB362263) | 5 | 0 | 0 | 0 | 6 | 0 | 0 | 0 | 4 | 0 | 0 | 0 |
| 115 | 14 | Muribaculum intestinale strain YL27 (NR 144616.1) | 5 | 0 | 0 | 0 | 6 | 0 | 0 | 0 | 3 | 0 | 0 | 0 |
| 153 | 14 | Acholeplasma parvum (T) H23M (AY538170) | 6 | 0 | 0 | 0 | 3 | 0 | 0 | 0 | 5 | 0 | 0 | 0 |
| 116 | 14 | Akkermansia muciniphila (T) Muc (AY271254) | 14 | 0 | 0 | 0 | 0 | 0 | 0 | 0 | 0 | 0 | 0 | 0 |
| 118 | 14 | Treponema caldarium (T) DSMZ7334 (EU580141) | 8 | 0 | 3 | 2 | 0 | 0 | 0 | 0 | 1 | 0 | 0 | 0 |
| 137 | 13 | [Clostridium] caenicola strain EBR596 (NR 126170.1) | 1 | 0 | 0 | 0 | 0 | 0 | 0 | 0 | 11 | 1 | 0 | 0 |
| 135 | 13 | Gracilibacter thermotolerans (T) JW/YJL-S1 (DQ117465) | 1 | 0 | 0 | 0 | 3 | 0 | 0 | 0 | 9 | 0 | 0 | 0 |
| 154 | 13 | Thermovirga lienii (T) Cas60314 (DQ071273) | 1 | 0 | 0 | 0 | 7 | 0 | 0 | 0 | 5 | 0 | 0 | 0 |
| 170 | 12 | Methanobacterium oryzae (T) FPi (AF028690) | 2 | 0 | 0 | 0 | 8 | 0 | 0 | 0 | 2 | 0 | 0 | 0 |
| 120 | 12 | Muribaculum intestinale strain YL27 (NR 144616.1) | 5 | 0 | 0 | 0 | 4 | 0 | 0 | 0 | 3 | 0 | 0 | 0 |
| 119 | 12 | Vallitalea pronyensis FatNI3 (KC876639) | 0 | 0 | 0 | 0 | 0 | 0 | 0 | 0 | 0 | 12 | 0 | 0 |
| 140 | 12 | Paracoccus aestuarii (T) B7 (EF660757) | 5 | 0 | 0 | 0 | 4 | 0 | 0 | 0 | 3 | 0 | 0 | 0 |
| 130 | 12 | Desulfuribacillus alkaliarsenatis AHT28 (HM046584) | 1 | 0 | 0 | 0 | 1 | 0 | 0 | 0 | 10 | 0 | 0 | 0 |
| 128 | 12 | Lactobacillus faecis AFL13-2 (AB812750) | 0 | 0 | 0 | 0 | 7 | 0 | 0 | 0 | 5 | 0 | 0 | 0 |
| 125 | 11 | Culturomica massiliensis strain Marseille-P2698 (NR 144745.1) | 0 | 0 | 0 | 0 | 9 | 0 | 0 | 0 | 2 | 0 | 0 | 0 |
| 127 | 11 | Desulfosporosinus orientis (T) DSM 765 (Y11570) | 0 | 0 | 0 | 0 | 0 | 0 | 0 | 0 | 11 | 0 | 0 | 0 |
| 146 | 11 | Smithella propionica (T) LYP (AF126282) | 9 | 0 | 0 | 0 | 1 | 0 | 0 | 0 | 1 | 0 | 0 | 0 |
| 142 | 10 | Bellilinea caldifistulae (T) GOMI-1 (AB243672) | 0 | 0 | 0 | 0 | 4 | 0 | 0 | 0 | 6 | 0 | 0 | 0 |
| 144 | 10 | Porphyromonas pogonae strain MI 10-1288 (NR 136443.1) | 5 | 0 | 0 | 0 | 0 | 0 | 0 | 0 | 5 | 0 | 0 | 0 |
| 205 | 10 | Natronoflexus pectinivorans AP1 (GQ922844) | 3 | 0 | 0 | 0 | 2 | 0 | 0 | 0 | 5 | 0 | 0 | 0 |
| 186 | 10 | Desulfitobacterium hafniense (T) DCB-2 (CP001336) | 3 | 0 | 0 | 0 | 0 | 0 | 0 | 0 | 7 | 0 | 0 | 0 |
| 141 | 10 | Soehngenia saccharolytica (T) BOR-Y (AY353956) | 4 | 0 | 0 | 0 | 2 | 0 | 0 | 0 | 4 | 0 | 0 | 0 |
| 179 | 10 | Caloramator fervidus (T) RT4. B1 (L09187) | 0 | 0 | 0 | 0 | 9 | 0 | 0 | 0 | 1 | 0 | 0 | 0 |
| 150 | 10 | Acholeplasma parvum (T) H23M (AY538170) | 1 | 0 | 0 | 0 | 2 | 0 | 0 | 0 | 7 | 0 | 0 | 0 |
| 167 | 9 | Cytophaga fermentans (T) ATCC 19072 (M58766) | 0 | 0 | 0 | 0 | 5 | 0 | 0 | 0 | 4 | 0 | 0 | 0 |
| 183 | 9 | Desulfovibrio psychrotolerans (T) type strain: PWC = JS1 (AM418397) | 7 | 0 | 0 | 0 | 1 | 0 | 0 | 0 | 1 | 0 | 0 | 0 |
| 177 | 9 | Muribaculum intestinale strain YL27 (NR 144616.1) | 2 | 0 | 0 | 0 | 2 | 0 | 0 | 0 | 5 | 0 | 0 | 0 |
| 164 | 9 | Thermanaerovibrio acidaminovorans strain DSM 6589 (NR 114455.1) | 0 | 0 | 0 | 0 | 9 | 0 | 0 | 0 | 0 | 0 | 0 | 0 |
| 159 | 9 | * | 3 | 0 | 0 | 0 | 4 | 0 | 0 | 0 | 2 | 0 | 0 | 0 |
| 156 | 9 | Moorella glycerini (T) YS6 (U82327) | 0 | 0 | 0 | 0 | 3 | 0 | 0 | 0 | 6 | 0 | 0 | 0 |
| 138 | 9 | Gracilibacter thermotolerans (T) JW/YJL-S1 (DQ117465) | 0 | 0 | 0 | 0 | 0 | 0 | 0 | 0 | 0 | 9 | 0 | 0 |
| 152 | 9 | Geovibrio ferrireducens (T) PAL-1 (X95744) | 3 | 0 | 0 | 0 | 5 | 0 | 0 | 0 | 1 | 0 | 0 | 0 |
| 149 | 9 | Anoxynatronum sibiricum (T) Z-7981 (AF522323) | 7 | 0 | 0 | 0 | 1 | 0 | 0 | 0 | 1 | 0 | 0 | 0 |
| 139 | 8 | Christensenella minuta YIT 12065 (AB490809) | 8 | 0 | 0 | 0 | 0 | 0 | 0 | 0 | 0 | 0 | 0 | 0 |
| 171 | 8 | Muribaculum intestinale strain YL27 (NR 144616.1) | 2 | 0 | 0 | 0 | 3 | 0 | 0 | 0 | 3 | 0 | 0 | 0 |
| 134 | 8 | * | 3 | 0 | 0 | 0 | 3 | 0 | 0 | 0 | 2 | 0 | 0 | 0 |
| 157 | 8 | Petrimonas sulfuriphila (T) BN3 (AY570690) | 7 | 0 | 0 | 0 | 0 | 0 | 0 | 0 | 1 | 0 | 0 | 0 |
| 143 | 7 | Methanobacterium subterraneum (T) A8p, DSM 11074 (X99044) | 0 | 0 | 0 | 0 | 0 | 0 | 0 | 0 | 0 | 7 | 0 | 0 |
| 220 | 7 | Alkaliphilus peptidifermentans (T) Z-7036 (EF382660) | 4 | 0 | 0 | 0 | 1 | 0 | 0 | 0 | 2 | 0 | 0 | 0 |
| 184 | 7 | Smithella propionica (T) LYP (AF126282) | 2 | 0 | 0 | 0 | 1 | 0 | 0 | 0 | 4 | 0 | 0 | 0 |
| 221 | 7 | Ornatilinea apprima P3M-1 (JQ292916) | 3 | 0 | 0 | 0 | 3 | 0 | 0 | 0 | 1 | 0 | 0 | 0 |
| 207 | 7 | Dethiobacter alkaliphilus (T) AHT 1 (EF422412) | 0 | 0 | 0 | 0 | 3 | 0 | 0 | 0 | 4 | 0 | 0 | 0 |
| 185 | 7 | Acetanaerobacterium elongatum (T) Z7 (AY487928) | 2 | 0 | 0 | 0 | 1 | 0 | 0 | 0 | 4 | 0 | 0 | 0 |
| 136 | 7 | anaerobic bacterium MO-CFX1 (AB598277) | 0 | 0 | 0 | 0 | 0 | 0 | 0 | 0 | 7 | 0 | 0 | 0 |
| 155 | 7 | Muribaculum intestinale strain YL27 (NR 144616.1) | 0 | 0 | 0 | 0 | 5 | 0 | 0 | 0 | 2 | 0 | 0 | 0 |
| 158 | 7 | Aminobacterium colombiense strain DSM 12261 (NR 074624.1) | 4 | 0 | 0 | 0 | 2 | 0 | 0 | 0 | 1 | 0 | 0 | 0 |
| 151 | 7 | * | 1 | 0 | 0 | 0 | 5 | 0 | 0 | 0 | 1 | 0 | 0 | 0 |
| 169 | 6 | Thermoanaerobacter thermocopriae (T) JT-3T (L09167) | 6 | 0 | 0 | 0 | 0 | 0 | 0 | 0 | 0 | 0 | 0 | 0 |
| 160 | 6 | Calditerricola yamamurae (T) YMO722 (AB308475) | 3 | 0 | 0 | 0 | 1 | 0 | 0 | 0 | 2 | 0 | 0 | 0 |
| 165 | 6 | Thermanaerothrix daxensis strain GNS-1 (NR 117865.1) | 2 | 0 | 0 | 0 | 4 | 0 | 0 | 0 | 0 | 0 | 0 | 0 |
| 161 | 6 | Desulfuromonas thiophila (T) NZ27 (DSMZ 8987) (Y11560) | 5 | 0 | 0 | 0 | 1 | 0 | 0 | 0 | 0 | 0 | 0 | 0 |
| 162 | 6 | Citrobacter sedlakii (T) CDC 4696-86 (AF025364) | 4 | 0 | 0 | 0 | 1 | 0 | 0 | 0 | 1 | 0 | 0 | 0 |
| 176 | 6 | Sedimentibacter saalensis (T) ZF2 (AJ404680) | 1 | 0 | 0 | 0 | 3 | 0 | 0 | 0 | 2 | 0 | 0 | 0 |
| 163 | 6 | Alkaliphilus crotonatoxidans (T) B11-2 (AF467248) | 5 | 0 | 0 | 0 | 1 | 0 | 0 | 0 | 0 | 0 | 0 | 0 |
| 192 | 6 | Smithella propionica (T) LYP (AF126282) | 5 | 0 | 0 | 0 | 1 | 0 | 0 | 0 | 0 | 0 | 0 | 0 |
| 214 | 6 | Thermotogales bacterium MesG1Ag4.2.16S.B (HM003109) | 3 | 0 | 0 | 0 | 3 | 0 | 0 | 0 | 0 | 0 | 0 | 0 |
| 166 | 5 | Defluviitalea saccharophila (T) LIND6LT2 (HQ020487) | 1 | 0 | 0 | 0 | 0 | 0 | 0 | 0 | 4 | 0 | 0 | 0 |
| 219 | 5 | Streptomyces cinereorectus (T) NBRC 15395 (AB184646) | 0 | 0 | 0 | 0 | 5 | 0 | 0 | 0 | 0 | 0 | 0 | 0 |
| 206 | 5 | Moorella humiferrea (T) 64 FGQ (GQ872425) | 4 | 0 | 0 | 0 | 0 | 0 | 0 | 0 | 1 | 0 | 0 | 0 |
| 199 | 5 | Methanofollis tationis (T) DSM 2702 (AF095272) | 3 | 0 | 0 | 0 | 1 | 0 | 0 | 0 | 1 | 0 | 0 | 0 |
| 208 | 5 | Levilinea saccharolytica (T) KIBI-1 (AB109439) | 0 | 0 | 0 | 0 | 4 | 0 | 0 | 0 | 1 | 0 | 0 | 0 |
| 168 | 5 | Anaerobranca zavarzinii (T) JW/VK-KS5Y (EF190921) | 1 | 0 | 0 | 0 | 3 | 0 | 0 | 0 | 1 | 0 | 0 | 0 |
| 188 | 5 | Muribaculum intestinale strain YL27 (NR 144616.1) | 2 | 0 | 0 | 0 | 1 | 0 | 0 | 0 | 2 | 0 | 0 | 0 |
| 180 | 4 | Pleomorphomonas diazotrophica R5-392 (JQ346801) | 1 | 0 | 0 | 0 | 3 | 0 | 0 | 0 | 0 | 0 | 0 | 0 |
| 173 | 4 | Mesorhizobium camelthorni (T) CCNWXJ40-4 (EU169581) | 2 | 0 | 0 | 0 | 2 | 0 | 0 | 0 | 0 | 0 | 0 | 0 |
| 215 | 4 | Lutispora thermophila (T) EBR46 (AB186360) | 3 | 0 | 0 | 0 | 1 | 0 | 0 | 0 | 0 | 0 | 0 | 0 |
| 217 | 4 | uncultured candidate division BRC1 bacterium LD1-PA21 (AY114315) | 4 | 0 | 0 | 0 | 0 | 0 | 0 | 0 | 0 | 0 | 0 | 0 |
| 218 | 4 | Gelria glutamica (T) TGO (AF321086) | 0 | 0 | 0 | 0 | 4 | 0 | 0 | 0 | 0 | 0 | 0 | 0 |
| 187 | 4 | Pseudobacteroides cellulosolvens (L35517) | 0 | 0 | 0 | 0 | 0 | 0 | 0 | 0 | 4 | 0 | 0 | 0 |
| 209 | 4 | Cytophaga xylanolytica strain DSM 6779 (NR 117112.1) | 1 | 0 | 0 | 0 | 2 | 0 | 0 | 0 | 1 | 0 | 0 | 0 |
| 175 | 4 | Muribaculum intestinale strain YL27 (NR 144616.1) | 0 | 0 | 0 | 0 | 1 | 0 | 0 | 0 | 3 | 0 | 0 | 0 |
| 182 | 4 | Desulfotomaculum acetoxidans (T) DSM 771 (Y11566) | 1 | 0 | 0 | 0 | 2 | 0 | 0 | 0 | 1 | 0 | 0 | 0 |
| 178 | 4 | Smithella propionica (T) LYP (AF126282) | 2 | 0 | 0 | 0 | 2 | 0 | 0 | 0 | 0 | 0 | 0 | 0 |
| 196 | 3 | Muribaculum intestinale strain YL27 (NR 144616.1) | 1 | 0 | 0 | 0 | 0 | 0 | 0 | 0 | 2 | 0 | 0 | 0 |
| 181 | 3 | * | 0 | 0 | 1 | 0 | 0 | 0 | 1 | 0 | 0 | 0 | 1 | 0 |
| 204 | 3 | Clostridium saccharolyticum (T) DSM 2544 (Y18185) | 0 | 0 | 0 | 0 | 0 | 0 | 0 | 0 | 3 | 0 | 0 | 0 |
| 193 | 3 | Candidatus Procabacter acanthamoebae (AF177427) | 2 | 0 | 0 | 0 | 1 | 0 | 0 | 0 | 0 | 0 | 0 | 0 |
| 191 | 3 | * | 3 | 0 | 0 | 0 | 0 | 0 | 0 | 0 | 0 | 0 | 0 | 0 |
| 174 | 3 | Muribaculum intestinale strain YL27 (NR 144616.1) | 2 | 0 | 0 | 0 | 0 | 0 | 0 | 0 | 1 | 0 | 0 | 0 |
| 202 | 3 | Methanoregula formicica (T) SMSP (AB479390) | 3 | 0 | 0 | 0 | 0 | 0 | 0 | 0 | 0 | 0 | 0 | 0 |
| 224 | 3 | Gracilibacter thermotolerans (T) JW/YJL-S1 (DQ117465) | 0 | 0 | 0 | 0 | 2 | 0 | 0 | 0 | 1 | 0 | 0 | 0 |
| 213 | 3 | uncultured bacterium FW34 (AF523981) | 3 | 0 | 0 | 0 | 0 | 0 | 0 | 0 | 0 | 0 | 0 | 0 |
| 223 | 3 | uncultured bacterium FW34 (AF523981) | 3 | 0 | 0 | 0 | 0 | 0 | 0 | 0 | 0 | 0 | 0 | 0 |
| 225 | 3 | Muribaculum intestinale strain YL27 (NR 144616.1) | 0 | 0 | 0 | 0 | 1 | 0 | 0 | 0 | 2 | 0 | 0 | 0 |
| 189 | 2 | Dehalobacter restrictus (T) PER-K23 (U84497) | 2 | 0 | 0 | 0 | 0 | 0 | 0 | 0 | 0 | 0 | 0 | 0 |
| 216 | 2 | Bacteroides acidifaciens (T) A40 (AB021164) | 1 | 0 | 0 | 0 | 1 | 0 | 0 | 0 | 0 | 0 | 0 | 0 |
| 194 | 2 | Saccharofermentans acetigenes (T) P6 (AY949857) | 2 | 0 | 0 | 0 | 0 | 0 | 0 | 0 | 0 | 0 | 0 | 0 |
| 190 | 2 | Desulfurispora thermophila (T) RA50E1 (AY548776) | 0 | 0 | 0 | 0 | 2 | 0 | 0 | 0 | 0 | 0 | 0 | 0 |
| 211 | 2 | Hydrogenophaga defluvii (T) type strain: BSB 9.5 (AJ585993) | 0 | 0 | 0 | 0 | 0 | 0 | 0 | 0 | 2 | 0 | 0 | 0 |
| 210 | 2 | Ruminococcaceae bacterium ZWB 4 (HG003571) | 0 | 0 | 0 | 0 | 1 | 0 | 0 | 0 | 1 | 0 | 0 | 0 |
| 222 | 2 | Candidatus Cloacimonas acidaminovorans str. Evry (CU466930) | 2 | 0 | 0 | 0 | 0 | 0 | 0 | 0 | 0 | 0 | 0 | 0 |
| 200 | 2 | Clostridium lavalense (T) CCRI-9842 (EF564277) | 2 | 0 | 0 | 0 | 0 | 0 | 0 | 0 | 0 | 0 | 0 | 0 |
| 203 | 2 | uncultured soil bacterium PBS-III-27 (AJ390456) | 0 | 0 | 0 | 0 | 2 | 0 | 0 | 0 | 0 | 0 | 0 | 0 |
| 198 | 2 | Acinetobacter indicus (T) A648 (HM047743) | 0 | 0 | 0 | 0 | 0 | 0 | 0 | 0 | 2 | 0 | 0 | 0 |
| 197 | 2 | Ornatilinea apprima P3M-1 (JQ292916) | 0 | 0 | 0 | 0 | 1 | 0 | 0 | 0 | 1 | 0 | 0 | 0 |
