# Supplementary material for: Kelpie: generating full-length ‘amplicons’ from whole-metagenome datasets
Source: PeerJ. 2019 Jan 30;6:e6174. doi: 10.7717/peerj.6174 (PMC6359901; doi:10.7717/peerj.6174)
Supplement: Table S4 — The #strain column is the stated number of strains present in the WGS reads, and the Abundance column is the total abundance for the specified organism (including all its strains). These tables are derived from the Excel spreadsheet ‘Kelpie - CAMI Low.xlsx’ which is available as . Part (A) includes all organisms, and Part (B) excludes those organisms that were found not to have a 16S V4 region in their assembled contigs. [file peerj-07-6174-s004.docx]

Table S4a: All stated organisms in CAMI Low Complexity dataset, with presence in assembled contigs and Kelpie extended amplicons. The #strns column is the stated number of strains present in the WGS reads, and the Abnd. column is the total abundance for the specified organism (including all its strains). This table is derived from the Excel spreadsheet ‘Kelpie - CAMI Low.xlsx’ which is available as Supplemental Table S7.

| **CAMI Low Complexity Gold Profile** |  |  |  | **Extracted v4 region from contigs** |  | **Kelpie profile** |  |
| --- | --- | --- | --- | --- | --- | --- | --- |
| **Lineage** | **species** | **#strns** | **Abnd.** | **Species/strain** | **Cov%** | **Species/strain** | **Abnd.** |
| Bacteria; Firmicutes; Negativicutes; Selenomonadales; Veillonellaceae; Schwartzia | Schwartzia succinivorans | 1 | 23.9% | Schwartzia succinivorans strain S1-1 (NR 029325.1) | 100 | Schwartzia succinivorans strain S1-1 (NR 029325.1) | 26.3% |
| Bacteria; Bacteroidetes; Sphingobacteriia; Sphingobacteriales; Chitinophagaceae; Hydrotalea | Hydrotalea sandarakina | 6 | 16.8% | Hydrotalea sandarakina strain AF-51 (NR 109380.1) | 100 | Hydrotalea sandarakina strain AF-51 (NR 109380.1) | 32.5% |
| Bacteria; Actinobacteria; Actinobacteria; Actinomycetales; Intrasporangiaceae; Tetrasphaera | Tetrasphaera duodecadis | 1 | 12.6% | Tetrasphaera duodecadis strain IAM 14868 (NR 040880.1) | 100 | Tetrasphaera duodecadis strain IAM 14868 (NR 040880.1) | 10.9% |
| Bacteria; Tenericutes; Mollicutes; Anaeroplasmatales; Anaeroplasmataceae; Anaeroplasma | Anaeroplasma bactoclasticum | 4 | 9.9% | *v4 region not present/complete in contigs* |  | *no WGS reads* |  |
| Bacteria; Firmicutes; Bacilli; Bacillales; Bacillales_f; Bacillales_g | Bacillales sp | 5 | 7.8% | Exiguobacterium acetylicum strain DSM 20416 (NR 043479.1) | 100 | Exiguobacterium acetylicum strain DSM 20416 (NR 043479.1) | 6.5% |
|  |  |  |  |  |  | Exiguobacterium sibiricum strain 255-15 (NR 075006.1) | 0.7% |
| Bacteria; Proteobacteria; Betaproteobacteria; Burkholderiales; Oxalobacteraceae; Janthinobacterium | Janthinobacterium sp | 2 | 6.6% | Massilia namucuonensis strain 333-1-0411 (NR 118215.1) | 100 | Massilia namucuonensis strain 333-1-0411 (NR 118215.1) | 5.6% |
| Bacteria; Proteobacteria; Gammaproteobacteria; Pseudomonadales; Pseudomonadaceae; Pseudomonas | Pseudomonas aeruginosa | 1 | 5.1% | Pseudomonas aeruginosa strain DSM 50071 (NR 117678.1) | 100 | Pseudomonas aeruginosa strain DSM 50071 (NR 117678.1) | 4.3% |
|  |  |  |  |  |  | Pseudomonas indica strain IMT37 (NR 028801.1) | 0.2% |
|  |  |  |  |  |  | Pseudomonas guangdongensis strain SgZ-6 (NR 118458.1) | 0.1% |
| Bacteria; Chloroflexi; Ktedonobacteria; Ktedonobacterales; Thermosporotrichaceae; Thermosporothrix | Thermosporothrix hazakensis | 1 | 4.7% | *v4 region not present/complete in contigs* |  | *no WGS reads* |  |
| Bacteria; Proteobacteria; Alphaproteobacteria; Rhodobacterales; Rhodobacteraceae; Paracoccus | Paracoccus denitrificans | 3 | 3.1% | Paracoccus denitrificans strain 381 (NR 026456.1) | 100 | Paracoccus denitrificans strain 381 (NR 026456.1) | 3.2% |
| Bacteria; Proteobacteria; Alphaproteobacteria; Rhodobacterales; Rhodobacteraceae; Defluviimonas | Defluviimonas denitrificans | 1 | 2.5% | Defluviimonas denitrificans strain D9-3 (NR 115019.1) | 100 | Defluviimonas denitrificans strain D9-3 (NR 115019.1) | 2.1% |
| Bacteria; Proteobacteria; Deltaproteobacteria; Desulfobacterales; Desulfobacteraceae; Desulfatibacillum | Desulfatibacillum alkenivorans | 1 | 1.6% | Desulfatibacillum alkenivorans strain PF2803 (NR 025795.1) | 100 | Desulfatibacillum alkenivorans strain PF2803 (NR 025795.1) | 1.8% |
| Bacteria; Actinobacteria; Actinobacteria; Actinomycetales; Actinomycetales_f; Actinomycetales_g | Actinomycetales sp | 1 | 0.9% | Williamsia phyllosphaerae strain C7 (NR 108495.1) | 100 | Williamsia phyllosphaerae strain C7 (NR 108495.1) | 0.3% |
| Bacteria; Bacteroidetes; Sphingobacteriia; Sphingobacteriales; Chitinophagaceae; Flavisolibacter | Flavisolibacter ginsengiterrae | 1 | 0.9% | *v4 region not present/complete in contigs (identical in V4 to next strain)* |  | *no WGS reads* |  |
| Bacteria; Bacteroidetes; Sphingobacteriia; Sphingobacteriales; Chitinophagaceae; Flavisolibacter | Flavisolibacter ginsengisoli | 1 | 0.6% | Flavisolibacter ginsengisoli strain Gsoil 643 (NR 041500.1) | 100 | Flavisolibacter ginsengisoli strain Gsoil 643 (NR 041500.1) | 3.6% |
| Bacteria; Proteobacteria; Betaproteobacteria; Neisseriales; Chromobacteriaceae; Andreprevotia | Andreprevotia lacus | 1 | 0.6% | *v4 region not present/complete in contigs* |  | *no WGS reads* |  |
| Bacteria; Firmicutes; Clostridia; Clostridiales; Peptostreptococcaceae; Tepidibacter | Tepidibacter formicigenes | 1 | 0.6% | Tepidibacter formicigenes strain DV1184 (NR 029081.1) | 100 | Tepidibacter formicigenes strain DV1184 (NR 029081.1) | 0.5% |
| Bacteria; Proteobacteria; Alphaproteobacteria; Rhodobacterales; Rhodobacteraceae; Albidovulum | Albidovulum xiamenense | 1 | 0.4% | Albidovulum xiamenense strain YBY-7 (NR 118031.1) | 100 | Albidovulum xiamenense strain YBY-7 (NR 118031.1) | 0.0% |
| Bacteria; Proteobacteria; Gammaproteobacteria; Xanthomonadales; Xanthomonadaceae; Xylella | Xylella fastidiosa | 1 | 0.3% | Xylella fastidiosa strain PCE-FF (NR 041779.1) | 100 | Xylella fastidiosa strain PCE-FF (NR 041779.1) | 0.7% |
| Bacteria; Proteobacteria; Betaproteobacteria; Burkholderiales; Comamonadaceae; Lampropedia | Lampropedia hyalina | 1 | 0.3% | Lampropedia hyalina strain IAM 14890 (NR 040942.1) | 97 | *incomplete WGS coverage of region* |  |
| Bacteria; Proteobacteria; Gammaproteobacteria; Xanthomonadales; Xanthomonadaceae; Lysobacter | Lysobacter oryzae | 1 | 0.3% | Lysobacter oryzae strain YC6269 (NR 044484.1) | 100 | Lysobacter oryzae strain YC6269 (NR 044484.1) | 0.7% |
| Bacteria; Firmicutes; Clostridia; Clostridiales; Proteinivoraceae; Anaerobranca | Anaerobranca californiensis | 1 | 0.1% | Anaerobranca zavarzinii strain JW/VK-KS5Y (NR 044155.1) | 96 | *incomplete WGS coverage of region* |  |
| Bacteria; Firmicutes; Clostridia; Clostridiales; Clostridiaceae; Clostridium | Clostridium caminithermale | 1 | 0.1% | *v4 region not present/complete in contigs* |  | *no WGS reads* |  |
| Bacteria; Firmicutes; Clostridia; Clostridiales; Clostridiaceae; Anaerosporobacter | Anaerosporobacter mobilis | 1 | 0.1% | *v4 region not present/complete in contigs* |  | *no WGS reads* |  |
| Bacteria; Bacteroidetes; Flavobacteriia; Flavobacteriales; Flavobacteriaceae; Nonlabens | Nonlabens dokdonensis | 1 | 0.1% | Nonlabens dokdonensis (NR 102491.1) | 50 | *incomplete WGS coverage of region* |  |
| Bacteria; Proteobacteria; Gammaproteobacteria; Xanthomonadales; Xanthomonadaceae; Rhodanobacter | Rhodanobacter sp | 1 | 0.0% | *v4 region not present/complete in contigs* |  | *no WGS reads* |  |

Table S4b: CAMI Low Complexity dataset showing organisms with **extracted V4 regions only**. Stated abundance shown for CAMI dataset, and folded relative abundance for Kelpie amplicons. The folded abundances are the sums for all strains for a given organism, and are given both as number of extended reads mapped and a percentage of all the Kelpie-generated extended reads.

| **CAMI Low Complexity Gold Profile** | **Stated** | **Extracted v4 region from contigs** | **Kelpie profile** | **Folded** | **Folded** |
| --- | --- | --- | --- | --- | --- |
| **Species** | **Abnd.** | **Species/strain** | **Species/strain** | **Reads** | **Abund.** |
| Schwartzia succinivorans | 28.2% | Schwartzia succinivorans strain S1-1 (NR 029325.1) | Schwartzia succinivorans strain S1-1 (NR 029325.1) | 615 | 26.3% |
| Hydrotalea sandarakina | 19.8% | Hydrotalea sandarakina strain AF-51 (NR 109380.1) | Hydrotalea sandarakina strain AF-51 (NR 109380.1) | 759 | 32.5% |
| Tetrasphaera duodecadis | 14.9% | Tetrasphaera duodecadis strain IAM 14868 (NR 040880.1) | Tetrasphaera duodecadis strain IAM 14868 (NR 040880.1) | 255 | 10.9% |
| Bacillales_sp | 9.2% | Exiguobacterium acetylicum strain DSM 20416 (NR 043479.1) | Exiguobacterium acetylicum strain DSM 20416 (NR 043479.1) | 169 | 7.2% |
| Janthinobacterium_sp | 7.8% | Massilia namucuonensis strain 333-1-0411 (NR 118215.1) | Massilia namucuonensis strain 333-1-0411 (NR 118215.1) | 132 | 5.6% |
| Pseudomonas aeruginosa | 6.0% | Pseudomonas aeruginosa strain DSM 50071 (NR 117678.1) | Pseudomonas aeruginosa strain DSM 50071 (NR 117678.1) | 108 | 4.6% |
| Paracoccus denitrificans | 3.7% | Paracoccus denitrificans strain 381 (NR 026456.1) | Paracoccus denitrificans strain 381 (NR 026456.1) | 74 | 3.2% |
| Defluviimonas denitrificans | 3.0% | Defluviimonas denitrificans strain D9-3 (NR 115019.1) | Defluviimonas denitrificans strain D9-3 (NR 115019.1) | 48 | 2.1% |
| Desulfatibacillum alkenivorans | 1.9% | Desulfatibacillum alkenivorans strain PF2803 (NR 025795.1) | Desulfatibacillum alkenivorans strain PF2803 (NR 025795.1) | 42 | 1.8% |
| Actinomycetales_sp | 1.1% | Williamsia phyllosphaerae strain C7 (NR 108495.1) | Williamsia phyllosphaerae strain C7 (NR 108495.1) | 8 | 0.3% |
| Flavisolibacter ginsengisoli | 1.8% | Flavisolibacter ginsengisoli strain Gsoil 643 (NR 041500.1) | Flavisolibacter ginsengisoli strain Gsoil 643 (NR 041500.1) | 83 | 3.6% |
| Tepidibacter formicigenes | 0.7% | Tepidibacter formicigenes strain DV1184 (NR 029081.1) | Tepidibacter formicigenes strain DV1184 (NR 029081.1) | 11 | 0.5% |
| Albidovulum xiamenense | 0.4% | Albidovulum xiamenense strain YBY-7 (NR 118031.1) | Albidovulum xiamenense strain YBY-7 (NR 118031.1) | 1 | 0.0% |
| Xylella fastidiosa | 0.4% | Xylella fastidiosa strain PCE-FF (NR 041779.1) | Xylella fastidiosa strain PCE-FF (NR 041779.1) | 17 | 0.7% |
| Lampropedia hyalina | 0.4% | Lampropedia hyalina strain IAM 14890 (NR 040942.1) | *incomplete WGS coverage of region* |  |  |
| Lysobacter oryzae | 0.3% | Lysobacter oryzae strain YC6269 (NR 044484.1) | Lysobacter oryzae strain YC6269 (NR 044484.1) | 16 | 0.7% |
| Anaerobranca californiensis | 0.2% | Anaerobranca zavarzinii strain JW/VK-KS5Y (NR 044155.1) | *incomplete WGS coverage of region* |  |  |
| Nonlabens dokdonensis | 0.1% | Nonlabens dokdonensis (NR 102491.1) | *incomplete WGS coverage of region* |  |  |
