# Supplementary material for: Kelpie: generating full-length ‘amplicons’ from whole-metagenome datasets
Source: PeerJ. 2019 Jan 30;6:e6174. doi: 10.7717/peerj.6174 (PMC6359901; doi:10.7717/peerj.6174)
Supplement: Table S5 — The #strain column is the stated number of strains present in the WGS reads, and the Abundance column is the total abundance for the specified organism (including all its strains). These tables are derived from the Excel spreadsheet ‘Kelpie—CAMI Medium.xlsx’ which is available as Table S8. Part (A) includes all organisms, and Part (B) excludes those organisms that were found not to have a 16S V4 region in their assembled contigs. [file peerj-07-6174-s005.docx]

Table S5a: All stated organisms in CAMI Medium Complexity dataset, with presence in assembled contigs and Kelpie extended amplicons. The #strns column is the stated number of strains as present in the WGS reads, Abnd. is abundance, and the Cov% is the percentage of kMers in the extracted sequence that are covered by the WGS reads. The tables presented in this file are derived from the Excel spreadsheet ‘Kelpie - CAMI Medium.xlsx’ which is available as Supplemental Table S8 .

| **CAMI Medium Complexity Gold Profile** |  |  |  | **Extracted v4 region from contigs** |  | **Kelpie profile** |  |
| --- | --- | --- | --- | --- | --- | --- | --- |
| **Lineage** | **species** | **#strns** | **Abnd.** | **Species/strain** | **Cov%** | **Species/strain** | **Abnd.** |
| Bacteria; Firmicutes; Clostridia; Thermoanaerobacterales; Thermoanaerobacteraceae; Moorella | Moorella thermoacetica | 2 | 9.5% | Moorella thermoautotrophica strain JW 701/3 (NR 029144.1) | 100 | Moorella thermoautotrophica strain JW 701/3 (NR 029144.1) | 17.4% |
| Bacteria; Proteobacteria; Alphaproteobacteria; Rhizobiales; Aurantimonadaceae; Aurantimonadaceae_g | Aurantimonadaceae sp | 2 | 8.5% | Aureimonas ureilytica strain 5715S-12 (NR 043995.1) | 100 | Aureimonas ureilytica strain 5715S-12 (NR 043995.1) | 7.7% |
| Bacteria; Proteobacteria; Alphaproteobacteria; Rhizobiales; Aurantimonadaceae; Aurantimonadaceae_g | Aurantimonadaceae sp | 2 | 0.2% |  |  | Aureimonas phyllosphaerae strain L9-753 (NR 118389.1) | 0.1% |
| Bacteria; Proteobacteria; Alphaproteobacteria; Sphingomonadales; Sphingomonadaceae; Sphingomonas | Sphingomonas aestuarii | 1 | 6.7% | Sphingomonas aestuarii strain K4 (NR 044341.1) | 100 | Sphingomonas aestuarii strain K4 (NR 044341.1) | 7.4% |
| Bacteria; Proteobacteria; Alphaproteobacteria; Sphingomonadales; Sphingomonadaceae; Sphingomonas | Sphingomonas phyllosphaerae | 1 | 0.7% | Sphingomonas phyllosphaerae strain FA2 (NR 029111.1) | 100 | Sphingomonas phyllosphaerae strain FA2 (NR 029111.1) | 0.4% |
| Bacteria; Proteobacteria; Alphaproteobacteria; Sphingomonadales; Sphingomonadaceae; Sphingomonas | Sphingomonas sp | 3 | 1.0% | Sphingomonas starnbergensis strain 382 (NR 109485.1) | 100 | Sphingomonas starnbergensis strain 382 (NR 109485.1) | 0.9% |
| Bacteria; Proteobacteria; Alphaproteobacteria; Rhizobiales; Rhizobiaceae; Sinorhizobium | Sinorhizobium meliloti | 2 | 5.6% | Sinorhizobium meliloti strain LMG 6133 (NR 118988.1) | 100 | Sinorhizobium meliloti strain LMG 6133 (NR 118988.1) | 5.5% |
| Bacteria; Proteobacteria; Betaproteobacteria; Burkholderiales; Comamonadaceae; Comamonadaceae_g | Comamonadaceae sp | 6 | 0.4% | Variovorax paradoxus strain 13-0-1D (NR 036930.1) | 100 | Variovorax paradoxus strain 13-0-1D (NR 036930.1) | 0.3% |
| Bacteria; Proteobacteria; Betaproteobacteria; Burkholderiales; Comamonadaceae; Comamonadaceae_g | Comamonadaceae sp |  | 2.4% | Limnohabitans parvus strain II-B4 (NR 125542.1) | 100 | Limnohabitans parvus strain II-B4 (NR 125542.1) | 3.7% |
| Bacteria; Proteobacteria; Betaproteobacteria; Burkholderiales; Comamonadaceae; Comamonadaceae_g | Comamonadaceae sp |  | 1.4% | Variovorax boronicumulans strain BAM-48 (NR 041588.1) | 100 | Variovorax boronicumulans strain BAM-48 (NR 041588.1) | 1.2% |
| Bacteria; Proteobacteria; Betaproteobacteria; Burkholderiales; Comamonadaceae; Comamonadaceae_g | Comamonadaceae sp |  | 0.6% | Piscinibacter aquaticus strain IMCC1728 (NR 043921.1) | 100 | Piscinibacter aquaticus strain IMCC1728 (NR 043921.1) | 0.3% |
| Bacteria; Proteobacteria; Betaproteobacteria; Burkholderiales; Comamonadaceae; Comamonadaceae_g | Comamonadaceae sp |  | 0.4% |  |  | Acidovorax anthurii (NR 041947.1) | 0.1% |
| Bacteria; Proteobacteria; Betaproteobacteria; Burkholderiales; Comamonadaceae; Comamonadaceae_g | Comamonadaceae sp |  | 0.2% |  |  | Albidiferax ferrireducens strain T118 (NR 074760.1) | 0.0% |
| Bacteria; Proteobacteria; Deltaproteobacteria; Desulfovibrionales; Desulfovibrionaceae; Desulfovibrio | Desulfovibrio bizertensis | 1 | 4.9% | Desulfovibrio bizertensis strain MB3 (NR 043808.1) | 100 | Desulfovibrio bizertensis strain MB3 (NR 043808.1) | 1.9% |
| Bacteria; Proteobacteria; Deltaproteobacteria; Desulfovibrionales; Desulfovibrionaceae; Desulfovibrio | Desulfovibrio alkalitolerans | 1 | 0.9% | Desulfovibrio alkalitolerans strain RT2 (NR 043069.1) | 100 | Desulfovibrio alkalitolerans strain RT2 (NR 043069.1) | 1.8% |
| Bacteria; Proteobacteria; Deltaproteobacteria; Desulfovibrionales; Desulfovibrionaceae; Desulfovibrio | Desulfovibrio vietnamensis | 1 | 0.0% | *v4 region not present/complete in contigs* |  | *no WGS reads* |  |
| Bacteria; Actinobacteria; Actinobacteria; Corynebacteriales; Nocardiaceae; Rhodococcus | Rhodococcus sp | 1 | 4.5% | Rhodococcus yunnanensis strain YIM 70056 (NR 043009.1) | 100 | Rhodococcus yunnanensis strain YIM 70056 (NR 043009.1) | 5.1% |
| Bacteria; Actinobacteria; Actinobacteria; Micrococcales; Cellulomonadaceae; Cellulomonas | Cellulomonas sp | 4 | 4.0% | Cellulomonas aerilata strain 5420S-23 (NR 044526.1) | 100 | Cellulomonas aerilata strain 5420S-23 (NR 044526.1) | 6.5% |
| Bacteria; Actinobacteria; Actinobacteria; Micrococcales; Cellulomonadaceae; Cellulomonas | Cellulomonas sp |  |  |  |  | Cellulomonas xylanilytica strain XIL11 (NR 029095.1) | 0.0% |
| Bacteria; Actinobacteria; Actinobacteria; Micrococcales; Cellulomonadaceae; Cellulomonas | Cellulomonas terrae | 1 | 0.0% | *v4 region not present/complete in contigs* |  | *no WGS reads* |  |
| Bacteria; Firmicutes; Clostridia; Clostridiales; Clostridiales Family XVI. Incertae Sedis; Carboxydocella | Carboxydocella sporoproducens | 6 | 3.0% | *v4 region not present/complete in contigs* |  | *no WGS reads* |  |
| Bacteria; Firmicutes; Clostridia; Clostridiales; Clostridiales Family XIX. Incertae Sedis; Acetoanaerobium | Acetoanaerobium noterae | 5 | 2.3% | *v4 region not present/complete in contigs* |  | *no WGS reads* |  |
| Bacteria; Proteobacteria; Alphaproteobacteria; Rhodospirillales; Rhodospirillaceae; Azospirillum | Azospirillum brasilense | 1 | 2.3% | *v4 region not present/complete in contigs* |  | *no WGS reads* |  |
| Bacteria; Proteobacteria; Alphaproteobacteria; Rhodospirillales; Rhodospirillaceae; Phaeospirillum | Phaeospirillum fulvum | 4 | 2.1% | Phaeospirillum molischianum strain DSM 120 (NR 104755.1) | 100 | Phaeospirillum molischianum strain DSM 120 (NR 104755.1) | 2.1% |
| Bacteria; Actinobacteria; Actinobacteria; Micrococcales; Cellulomonadaceae; Oerskovia | Oerskovia sp | 2 | 2.1% | Sanguibacter marinus strain 1-19 (NR 042311.1) | 100 | Sanguibacter marinus strain 1-19 (NR 042311.1) | 2.3% |
| Bacteria; Actinobacteria; Actinobacteria; Propionibacteriales; Nocardioidaceae; Aeromicrobium | Aeromicrobium ponti | 3 | 2.1% | Aeromicrobium ponti strain HSW-1 (NR 042659.1) | 100 | Aeromicrobium ponti strain HSW-1 (NR 042659.1) | 1.5% |
| Bacteria; Proteobacteria; Alphaproteobacteria; Rhizobiales; Bradyrhizobiaceae; Bosea | Bosea sp | 2 | 1.9% | Bosea vaviloviae strain Vaf-18 (NR 136423.1) | 100 | Bosea vaviloviae strain Vaf-18 (NR 136423.1) | 1.4% |
| Bacteria; Proteobacteria; Betaproteobacteria; Burkholderiales; Oxalobacteraceae; Massilia | uncultured Massilia sp. | 4 | 1.9% | Massilia niabensis strain 5420S-26 (NR 044571.1) | 100 | Massilia niabensis strain 5420S-26 (NR 044571.1) | 3.1% |
| Bacteria; Proteobacteria; Gammaproteobacteria; Alteromonadales; Ferrimonadaceae; Ferrimonas | Ferrimonas marina | 1 | 1.8% | *v4 region not present/complete in contigs* |  | *no WGS reads* |  |
| Bacteria; Actinobacteria; Actinobacteria; Streptomycetales; Streptomycetaceae; Streptomycetaceae_g | Streptomycetaceae sp | 2 | 1.6% | Streptomyces lunaelactis strain MM109 (NR 134822.1) | 100 | Streptomyces lunaelactis strain MM109 (NR 134822.1) | 0.9% |
| Bacteria; Bacteroidetes; Flavobacteriia; Flavobacteriales; Flavobacteriaceae; Formosa | Formosa spongicola | 1 | 1.5% | Ichthyenterobacterium magnum strain Th6 (NR 134750.1) | 100 | Ichthyenterobacterium magnum strain Th6 (NR 134750.1) | 1.4% |
| Bacteria; Firmicutes; Negativicutes; Selenomonadales; Veillonellaceae; Propionispora | Propionispora hippei | 1 | 1.5% | Propionispora hippei strain KS (NR 036875.1) | 100 | Propionispora hippei strain KS (NR 036875.1) | 1.7% |
| Bacteria; Proteobacteria; Alphaproteobacteria; Rhizobiales; Phyllobacteriaceae; Aquamicrobium | *Aquamicrobium sp* | 2 | 1.5% | Mesorhizobium caraganae strain CCBAU 11299 (NR 044118.1) | 100 | Mesorhizobium caraganae strain CCBAU 11299 (NR 044118.1) | 2.0% |
| Bacteria; Proteobacteria; Alphaproteobacteria; Rhizobiales; Phyllobacteriaceae; Aquamicrobium | *Aquamicrobium sp* | 2 | 0.0% | Mesorhizobium chacoense strain PR5 (NR 025411.1) | 65 | *incomplete WGS coverage of region* |  |
| Bacteria; Firmicutes; Bacilli; Bacillales; Bacillales_f; Bacillales_g | Bacillales sp | 3 | 1.4% | Exiguobacterium acetylicum strain DSM 20416 (NR 043479.1) | 100 | Exiguobacterium acetylicum strain DSM 20416 (NR 043479.1) | 0.9% |
| Bacteria; Bacteroidetes; Flavobacteriia; Flavobacteriales; Flavobacteriaceae; Olleya | Olleya aquimaris | 1 | 1.4% | Olleya aquimaris strain L-4 (NR 104531.1) | 100 | Olleya aquimaris strain L-4 (NR 104531.1) | 6.8% |
| Bacteria; Proteobacteria; Alphaproteobacteria; Rhodobacterales; Rhodobacteraceae; Rhodobacter | Rhodobacter capsulatus | 2 | 1.3% | Rhodobacter viridis strain JA737 (NR 108854.1) | 100 | Rhodobacter viridis strain JA737 (NR 108854.1) | 1.7% |
| Archaea; Euryarchaeota; Halobacteria; Halobacteriales; Halobacteriaceae; Halobacterium | Halobacterium salinarum | 2 | 1.3% | Halobacterium salinarum strain 91-R6 (NR 025555.1) | 100 | Halobacterium salinarum strain 91-R6 (NR 025555.1) | 1.4% |
| Bacteria; Actinobacteria; Actinobacteria; Micrococcales; Intrasporangiaceae; Janibacter | Janibacter limosus | 1 | 1.1% | Knoellia locipacati strain DMZ1 (NR 109064.1) | 100 | Janibacter indicus strain 0704P10-1 (NR 134061.1) | 1.1% |
|  |  |  |  |  |  | Luteimicrobium album strain RI148-Li105 (NR 108122.1) | 0.2% |
| Archaea; Euryarchaeota; Thermoplasmata; Thermoplasmatales; Picrophilaceae; Picrophilus | Picrophilus oshimae | 1 | 1.1% | Picrophilus torridus strain DSM 9790 (NR 074187.1) | 100 | Picrophilus torridus strain DSM 9790 (NR 074187.1) | 1.4% |
| Bacteria; Proteobacteria; Gammaproteobacteria; Enterobacteriales; Enterobacteriaceae; Rahnella | Rahnella aquatilis | 1 | 0.9% | Serratia aquatilis strain 2015-2462-01 (NR 147771.1) | 100 | Serratia aquatilis strain 2015-2462-01 (NR 147771.1) | 0.9% |
| Bacteria; Proteobacteria; Betaproteobacteria; Burkholderiales; Comamonadaceae; Diaphorobacter | Diaphorobacter oryzae | 1 | 0.8% | Diaphorobacter oryzae strain RF3 (NR 044472.1) | 100 | Diaphorobacter oryzae strain RF3 (NR 044472.1) | 0.4% |
| Bacteria; Firmicutes; Clostridia; Thermoanaerobacterales; Thermoanaerobacteraceae; Thermoanaerobacter | Thermoanaerobacter thermohydrosulfuricus | 2 | 0.8% | *v4 region not present/complete in contigs* |  | *no WGS reads* |  |
| Bacteria; Firmicutes; Clostridia; Clostridiales; Peptostreptococcaceae; Tepidibacter | Tepidibacter thalassicus | 1 | 0.7% | Tepidibacter thalassicus strain SC 562 (NR 025678.1) | 100 | Tepidibacter thalassicus strain SC 562 (NR 025678.1) | 0.8% |
| Bacteria; Proteobacteria; Gammaproteobacteria; Pseudomonadales; Pseudomonadaceae; Azotobacter | Azotobacter vinelandii | 1 | 0.7% | *v4 region not present/complete in contigs* |  | *no WGS reads* |  |
| Bacteria; Actinobacteria; Actinobacteria; Micrococcales; Microbacteriaceae; Microbacteriaceae_g | Microbacteriaceae sp | 1 | 0.7% | Frondihabitans australicus strain E1HC-02 (NR 043897.1) | 100 | Frondihabitans australicus strain E1HC-02 (NR 043897.1) | 1.2% |
| Bacteria; Proteobacteria; Betaproteobacteria; Methylophilales; Methylophilaceae; Methylophilus | Methylophilus sp | 3 | 0.6% | Methylophilus flavus strain Ship (NR 104519.1) | 100 | Methylophilus flavus strain Ship (NR 104519.1) | 0.5% |
| Bacteria; Actinobacteria; Actinobacteria; Actinomycetales; Intrasporangiaceae; Phycicoccus | Phycicoccus sp | 2 | 0.6% | Phycicoccus dokdonensis strain DS-8 (NR 044286.1) | 100 | Phycicoccus dokdonensis strain DS-8 (NR 044286.1) | 0.3% |
| Bacteria; Firmicutes; Bacilli; Bacillales; Planococcaceae; Sporosarcina | Sporosarcina newyorkensis | 2 | 0.5% | *v4 region not present/complete in contigs* |  | *no WGS reads* |  |
| Bacteria; Proteobacteria; Gammaproteobacteria; Legionellales; Legionellaceae; Tatlockia | Legionella maceachernii | 1 | 0.4% | Legionella maceachernii strain ATCC 35300 (NR 041790.1) | 100 | Legionella maceachernii strain ATCC 35300 (NR 041790.1) | 0.7% |
| Bacteria; Firmicutes; Clostridia; Clostridiales; Clostridiaceae; Butyricicoccus | Butyricicoccus pullicaecorum | 1 | 0.4% | *v4 region not present/complete in contigs* |  | *no WGS reads* |  |
| Bacteria; Firmicutes; Clostridia; Clostridiales; Clostridiaceae; Caloranaerobacter | Caloranaerobacter azorensis | 1 | 0.4% | Caloranaerobacter azorensis strain MV1087 (NR 028919.1) | 73 | *incomplete WGS coverage of region* |  |
| Bacteria; Firmicutes; Clostridia; Clostridiales; Clostridiaceae; Clostridium | Clostridium grantii | 1 | 0.4% | Clostridium grantii strain A1 (NR 026131.1) | 94 | Clostridium grantii strain A1 (NR 026131.1) | 0.2% |
| Bacteria; Firmicutes; Clostridia; Clostridiales; Clostridiaceae; Clostridium | Clostridium tetani | 1 | 0.2% | Clostridium cochlearium strain ATCC 17787 (NR 044717.2) | 54 | *incomplete WGS coverage of region* |  |
| Bacteria; Firmicutes; Bacilli; Bacillales; Bacillaceae; Bacillus | Bacillus coagulans | 1 | 0.3% | Bacillus coagulans strain NBRC 12583 (NR 041523.1) | 90 | Bacillus coagulans strain NBRC 12583 (NR 041523.1) | 0.5% |
| Bacteria; Actinobacteria; Actinobacteria; Micrococcales; Promicromonosporaceae; Promicromonospora | Promicromonospora umidemergens | 1 | 0.3% | Promicromonospora vindobonensis strain V-45 (NR 042146.1) | 73 | Promicromonospora vindobonensis strain V-45 (NR 042146.1) | 1.1% |
| Bacteria; Actinobacteria; Actinobacteria; Acidimicrobiales; Acidimicrobiaceae; Ferrithrix | Ferrithrix thermotolerans | 1 | 0.3% | Ferrithrix thermotolerans strain Y005 (NR 042751.1) | 14 | *incomplete WGS coverage of region* |  |
| Bacteria; Actinobacteria; Actinobacteria; Corynebacteriales; Nocardiaceae; Nocardia | Nocardia g | 1 | 0.3% | Nocardia coubleae strain OFN N12 (NR 104567.1) | 100 | Nocardia coubleae strain OFN N12 (NR 104567.1) | 0.2% |
| Bacteria; Proteobacteria; Alphaproteobacteria; Rhodobacterales; Rhodobacteraceae; Paracoccus | Paracoccus pantotrophus | 2 | 0.3% | Paracoccus versutus strain ATCC 25364 (NR 042713.1) | 100 | Paracoccus versutus strain ATCC 25364 (NR 042713.1) | 0.7% |
| Bacteria; Proteobacteria; Alphaproteobacteria; Rhodobacterales; Rhodobacteraceae; Rhodobaca | Rhodobaca barguzinensis | 1 | 0.1% | Rhodobaca barguzinensis strain VKM B-2406 (NR 044285.1) | 92 | Rhodobaca barguzinensis strain VKM B-2406 (NR 044285.1) | 0.2% |
| Bacteria; Bacteroidetes; Sphingobacteriia; Sphingobacteriales; Sphingobacteriaceae; Pedobacter | Pedobacter sp | 1 | 0.3% | Pedobacter jejuensis strain THG-DR3 (NR 133810.1) | 84 | Pedobacter jejuensis strain THG-DR3 (NR 133810.1) | 0.3% |
| Bacteria; Bacteroidetes; Sphingobacteriia; Sphingobacteriales; Sphingobacteriaceae; Pedobacter | Pedobacter agri | 1 | 0.2% | Pedobacter ginsenosidimutans strain THG-45 (NR 108685.1) | 100 | Pedobacter ginsenosidimutans strain THG-45 (NR 108685.1) | 0.0% |
| Bacteria; Actinobacteria; Actinobacteria; Pseudonocardiales; Pseudonocardiaceae; Kibdelosporangium | Kibdelosporangium aridum | 1 | 0.3% | Kibdelosporangium aridum subsp. largum strain SKF-AAD-609 (NR 025571.1) | 100 | Kibdelosporangium aridum subsp. largum strain SKF-AAD-609 (NR 025571.1) | 0.4% |
| Bacteria; Tenericutes; Mollicutes; Acholeplasmatales; Acholeplasmataceae; Acholeplasma | Acholeplasma oculi | 1 | 0.3% | Acholeplasma oculi strain 19L (NR 025960.1) | 11 | *incomplete WGS coverage of region* |  |
| Bacteria; Proteobacteria; Betaproteobacteria; Nitrosomonadales; Nitrosomonadaceae; Nitrosomonas | Nitrosomonas europaea | 1 | 0.3% | Nitrosomonas europaea strain C-31 (NR 040879.1) | 96 | Nitrosomonas europaea strain C-31 (NR 040879.1) | 0.6% |
| Bacteria; Bacteroidetes; Sphingobacteriia; Sphingobacteriales; Sphingobacteriaceae; Sphingobacterium | Sphingobacterium psychroaquaticum | 1 | 0.3% | *v4 region not present/complete in contigs* |  | *no WGS reads* |  |
| Bacteria; Bacteroidetes; Sphingobacteriia; Sphingobacteriales; Sphingobacteriaceae; Sphingobacterium | Sphingobacterium nematocida | 1 | 0.1% | Sphingobacterium nematocida strain M-SX103 (NR 122101.1) | 34 | *incomplete WGS coverage of region* |  |
| Bacteria; Proteobacteria; Gammaproteobacteria; Vibrionales; Vibrionaceae; Vibrio | Vibrio cincinnatiensis | 1 | 0.2% | *v4 region not present/complete in contigs* |  | *no WGS reads* |  |
| Bacteria; Proteobacteria; Gammaproteobacteria; Vibrionales; Vibrionaceae; Vibrio | Vibrio gazogenes | 1 | 0.2% | *v4 region not present/complete in contigs* |  | *no WGS reads* |  |
| Bacteria; Actinobacteria; Actinobacteria; Pseudonocardiales; Pseudonocardiaceae; Lechevalieria | Lechevalieria flava | 1 | 0.2% | *v4 region not present/complete in contigs* |  | *no WGS reads* |  |
| Bacteria; Actinobacteria; Actinobacteria; Pseudonocardiales; Pseudonocardiaceae; Lechevalieria | Lechevalieria aerocolonigenes | 1 | 0.1% | *v4 region not present/complete in contigs* |  | *no WGS reads* |  |
| Bacteria; Firmicutes; Clostridia; Clostridiales; Clostridiaceae; Caminicella | Caminicella sporogenes | 1 | 0.2% | Caminicella sporogenes strain AM1114 (NR 025485.1) | 100 | *incomplete WGS coverage of region* |  |
| Bacteria; Firmicutes; Clostridia; Clostridiales; Peptococcaceae; Desulfotomaculum | Desulfotomaculum hydrothermale | 1 | 0.2% | *v4 region not present/complete in contigs* |  | *no WGS reads* |  |
| Bacteria; Firmicutes; Bacilli; Lactobacillales; Enterococcaceae; Tetragenococcus | Tetragenococcus halophilus | 1 | 0.2% | *v4 region not present/complete in contigs* |  | *no WGS reads* |  |
| Bacteria; Firmicutes; Clostridia; Clostridiales; Lachnospiraceae; Butyrivibrio | Butyrivibrio fibrisolvens | 1 | 0.2% | Butyrivibrio fibrisolvens strain ATCC 19171 (NR 025981.1) | 99 | Butyrivibrio fibrisolvens strain ATCC 19171 (NR 025981.1) | 0.3% |
| Bacteria; Firmicutes; Clostridia; Clostridiales; Lachnospiraceae; Butyrivibrio | Butyrivibrio hungatei | 1 | 0.2% | Butyrivibrio hungatei strain JK 615 (NR 025525.1) | 100 | Butyrivibrio hungatei strain JK 615 (NR 025525.1) | 0.2% |
| Bacteria; Actinobacteria; Actinobacteria; Propionibacteriales; Propionibacteriaceae; Tessaracoccus | Tessaracoccus bendigoensis | 1 | 0.2% | Tessaracoccus flavescens strain SST-39 (NR 042550.1) | 48 | *incomplete WGS coverage of region* |  |
| Bacteria; Bacteroidetes; Bacteroidia; Bacteroidales; Porphyromonadaceae; Porphyromonas | Porphyromonas crevioricanis | 1 | 0.2% | Porphyromonas crevioricanis strain NUM 402 (NR 104834.1) | 34 | *incomplete WGS coverage of region* |  |
| Bacteria; Firmicutes; Clostridia; Clostridiales; Peptostreptococcaceae; Intestinibacter | Intestinibacter bartlettii | 1 | 0.2% | *v4 region not present/complete in contigs* |  | *no WGS reads* |  |
| Bacteria; Proteobacteria; Alphaproteobacteria; Caulobacterales; Caulobacteraceae; Phenylobacterium | Phenylobacterium composti | 1 | 0.2% | Phenylobacterium zucineum strain HLK1 (NR 074119.1) | 100 | Phenylobacterium zucineum strain HLK1 (NR 074119.1) | 0.2% |
| Bacteria; Actinobacteria; Actinobacteria; Micrococcales; Microbacteriaceae; Curtobacterium | Curtobacterium sp. B20 | 1 | 0.2% | Curtobacterium flaccumfaciens strain LMG 3645 (NR 025467.1) | 81 | Curtobacterium flaccumfaciens strain LMG 3645 (NR 025467.1) | 0.2% |
| Bacteria; Proteobacteria; Alphaproteobacteria; Rhizobiales; Xanthobacteraceae; Xanthobacter | Xanthobacter autotrophicus | 1 | 0.1% | Xanthobacter autotrophicus strain 7c (NR 026308.1) | 93 | Xanthobacter autotrophicus strain 7c (NR 026308.1) | 0.2% |
| Bacteria; Actinobacteria; Actinobacteria; Corynebacteriales; Mycobacteriaceae; Mycobacterium | Mycobacterium sp | 1 | 0.1% | Mycobacterium obuense strain 47001 (NR 029218.1) | 29 | *incomplete WGS coverage of region* |  |
| Bacteria; Firmicutes; Clostridia; Clostridiales; Eubacteriaceae; Eubacterium | [Eubacterium] yurii | 1 | 0.1% | [Eubacterium] yurii strain SM14 (NR 104843.1) | 100 | *incomplete WGS coverage of region* |  |
| Bacteria; Proteobacteria; Alphaproteobacteria; Rhizobiales; Xanthobacteraceae; Pseudoxanthobacter | Pseudoxanthobacter soli | 1 | 0.1% | Pseudoxanthobacter soli strain CC4 (NR 044225.1) | 63 | *incomplete WGS coverage of region* |  |
| Bacteria; Bacteroidetes; Cytophagia; Cytophagales; Cytophagaceae; Ohtaekwangia | Ohtaekwangia koreensis | 1 | 0.1% | Ohtaekwangia koreensis strain 3B-2 (NR 117435.1) | 70 | *incomplete WGS coverage of region* |  |
| Bacteria; Bacteroidetes; Cytophagia; Cytophagales; Cytophagaceae; Ohtaekwangia | Ohtaekwangia kribbensis | 1 | 0.0% | Ohtaekwangia kribbensis strain 10AO (NR 117436.1) | 83 | *incomplete WGS coverage of region* |  |
| Bacteria; Bacteroidetes; Flavobacteriia; Flavobacteriales; Flavobacteriaceae; Chryseobacterium | Chryseobacterium sp | 1 | 0.1% | Chryseobacterium gregarium strain P 461/12 (NR 042647.1) | 19 | *incomplete WGS coverage of region* |  |
| Bacteria; Proteobacteria; Alphaproteobacteria; Rhodobacterales; Rhodobacteraceae; Roseinatronobacter | Roseinatronobacter thiooxidans | 1 | 0.0% | *v4 region not present/complete in contigs* |  | *no WGS reads* |  |
| Bacteria; Firmicutes; Clostridia; Clostridiales; Clostridiaceae; Lutispora | Lutispora thermophila | 1 | 0.0% | Lutispora thermophila strain EBR46 (NR 041236.1) | 54 | *incomplete WGS coverage of region* |  |
| Bacteria; Firmicutes; Bacilli; Bacillales; Paenibacillaceae; Paenibacillus | Paenibacillus frigoriresistens | 1 | 0.0% | Paenibacillus frigoriresistens strain YIM 016 (NR 109546.1) | 36 | *incomplete WGS coverage of region* |  |
| Bacteria; Proteobacteria; Alphaproteobacteria; Rhizobiales; Rhizobiaceae; Rhizobium | Rhizobium sp | 1 | 0.0% | Rhizobium daejeonense strain L61 (NR 042851.1) | 35 | *incomplete WGS coverage of region* |  |
| Bacteria; Firmicutes; Clostridia; Clostridiales; Eubacteriaceae; Garciella | Garciella nitratireducens | 1 | 0.0% | *v4 region not present/complete in contigs* |  | *no WGS reads* |  |
| Bacteria; Firmicutes; Bacilli; Bacillales; Staphylococcaceae; Salinicoccus | Salinicoccus kunmingensis | 1 | 0.0% | *v4 region not present/complete in contigs* |  | *no WGS reads* |  |
| Bacteria; Bacteroidetes; Bacteroidia; Bacteroidales; Prevotellaceae; Prevotella | Prevotella oulorum | 1 | 0.0% | Prevotella oulorum strain WPH 179 (NR 029147.1) | 0 | *incomplete WGS coverage of region* |  |
| Bacteria; Proteobacteria; Betaproteobacteria; Neisseriales; Neisseriaceae; Alysiella | Alysiella filiformis | 1 | 0.0% | Alysiella crassa strain IAM 14969 (NR 040932.1) | 11 | *incomplete WGS coverage of region* |  |

Table S5b: CAMI Medium Complexity dataset showing organisms with **extracted V4 regions only**. Stated abundance shown for CAMI dataset, and folded relative abundance for Kelpie amplicons. The folded abundances are the sums for all strains for a given organism, and are given both as number of extended reads mapped and a percentage of all the Kelpie-generated extended reads.

| **CAMI Medium Complexity Gold Profile** | **Stated** | **Extracted v4 region from contigs** |  | **Kelpie profile** | **Folded** | **Folded** |
| --- | --- | --- | --- | --- | --- | --- |
| **Species** | **Abnd.** | **Species/strain** | **Cov%** | **Species/strain** | **Reads** | **Abund.** |
| Moorella thermoacetica | 11.0% | Moorella thermoautotrophica strain JW 701/3 (NR 029144.1) | 100 | Moorella thermoautotrophica strain JW 701/3 (NR 029144.1) | 562 | 17.35% |
| Aurantimonadaceae_sp | 10.1% | Aureimonas ureilytica strain 5715S-12 (NR 043995.1) | 100 | Aureimonas ureilytica strain 5715S-12 (NR 043995.1) | 251 | 7.81% |
| Sphingomonas aestuarii | 7.8% | Sphingomonas aestuarii strain K4 (NR 044341.1) | 100 | Sphingomonas aestuarii strain K4 (NR 044341.1) | 240 | 7.41% |
| Sinorhizobium meliloti | 6.5% | Sinorhizobium meliloti strain LMG 6133 (NR 118988.1) | 100 | Sinorhizobium meliloti strain LMG 6133 (NR 118988.1) | 179 | 5.53% |
| Desulfovibrio bizertensis | 5.7% | Desulfovibrio bizertensis strain MB3 (NR 043808.1) | 100 | Desulfovibrio bizertensis strain MB3 (NR 043808.1) | 62 | 1.91% |
| Rhodococcus_sp | 5.2% | Rhodococcus yunnanensis strain YIM 70056 (NR 043009.1) | 100 | Rhodococcus yunnanensis strain YIM 70056 (NR 043009.1) | 166 | 5.13% |
| Cellulomonas_sp | 4.7% | Cellulomonas aerilata strain 5420S-23 (NR 044526.1) | 100 | Cellulomonas aerilata strain 5420S-23 (NR 044526.1) | 209 | 6.48% |
| Comamonadaceae_sp | 2.7% | Limnohabitans parvus strain II-B4 (NR 125542.1) | 100 | Limnohabitans parvus strain II-B4 (NR 125542.1) | 119 | 3.67% |
| Phaeospirillum fulvum | 2.5% | Phaeospirillum molischianum strain DSM 120 (NR 104755.1) | 100 | Phaeospirillum molischianum strain DSM 120 (NR 104755.1) | 69 | 2.13% |
| Oerskovia_sp | 2.4% | Sanguibacter marinus strain 1-19 (NR 042311.1) | 100 | Sanguibacter marinus strain 1-19 (NR 042311.1) | 76 | 2.35% |
| Aeromicrobium ponti | 2.4% | Aeromicrobium ponti strain HSW-1 (NR 042659.1) | 100 | Aeromicrobium ponti strain HSW-1 (NR 042659.1) | 47 | 1.45% |
| Bosea_sp | 2.2% | Bosea vaviloviae strain Vaf-18 (NR 136423.1) | 100 | Bosea vaviloviae strain Vaf-18 (NR 136423.1) | 45 | 1.39% |
| uncultured Massilia sp. | 2.2% | Massilia niabensis strain 5420S-26 (NR 044571.1) | 100 | Massilia niabensis strain 5420S-26 (NR 044571.1) | 100 | 3.09% |
| Streptomycetaceae_sp | 1.9% | Streptomyces lunaelactis strain MM109 (NR 134822.1) | 100 | Streptomyces lunaelactis strain MM109 (NR 134822.1) | 28 | 0.86% |
| Formosa spongicola | 1.8% | Ichthyenterobacterium magnum strain Th6 (NR 134750.1) | 100 | Ichthyenterobacterium magnum strain Th6 (NR 134750.1) | 45 | 1.39% |
| Propionispora hippei | 1.8% | Propionispora hippei strain KS (NR 036875.1) | 100 | Propionispora hippei strain KS (NR 036875.1) | 55 | 1.70% |
| *Aquamicrobium_sp* | 1.8% | Mesorhizobium caraganae strain CCBAU 11299 (NR 044118.1) | 100 | Mesorhizobium caraganae strain CCBAU 11299 (NR 044118.1) | 64 | 1.98% |
| Bacillales_sp | 1.6% | Exiguobacterium acetylicum strain DSM 20416 (NR 043479.1) | 100 | Exiguobacterium acetylicum strain DSM 20416 (NR 043479.1) | 28 | 0.86% |
| Comamonadaceae_sp | 1.6% | Variovorax boronicumulans strain BAM-48 (NR 041588.1) | 100 | Variovorax boronicumulans strain BAM-48 (NR 041588.1) | 38 | 1.17% |
| Olleya aquimaris | 1.6% | Olleya aquimaris strain L-4 (NR 104531.1) | 100 | Olleya aquimaris strain L-4 (NR 104531.1) | 219 | 6.76% |
| Rhodobacter capsulatus | 1.5% | Rhodobacter viridis strain JA737 (NR 108854.1) | 100 | Rhodobacter viridis strain JA737 (NR 108854.1) | 54 | 1.67% |
| Halobacterium salinarum | 1.5% | Halobacterium salinarum strain 91-R6 (NR 025555.1) | 100 | Halobacterium salinarum strain 91-R6 (NR 025555.1) | 45 | 1.39% |
| Janibacter limosus | 1.3% | Knoellia locipacati strain DMZ1 (NR 109064.1) | 100 | Janibacter indicus strain 0704P10-1 (NR 134061.1) | 37 | 1.30% |
| Picrophilus oshimae | 1.3% | Picrophilus torridus strain DSM 9790 (NR 074187.1) | 100 | Picrophilus torridus strain DSM 9790 (NR 074187.1) | 45 | 1.39% |
| Sphingomonas_sp | 1.1% | Sphingomonas starnbergensis strain 382 (NR 109485.1) | 100 | Sphingomonas starnbergensis strain 382 (NR 109485.1) | 30 | 0.93% |
| Desulfovibrio alkalitolerans | 1.1% | Desulfovibrio alkalitolerans strain RT2 (NR 043069.1) | 100 | Desulfovibrio alkalitolerans strain RT2 (NR 043069.1) | 58 | 1.79% |
| Rahnella aquatilis | 1.0% | Serratia aquatilis strain 2015-2462-01 (NR 147771.1) | 100 | Serratia aquatilis strain 2015-2462-01 (NR 147771.1) | 29 | 0.90% |
| Diaphorobacter oryzae | 1.0% | Diaphorobacter oryzae strain RF3 (NR 044472.1) | 100 | Diaphorobacter oryzae strain RF3 (NR 044472.1) | 14 | 0.43% |
| Tepidibacter thalassicus | 0.9% | Tepidibacter thalassicus strain SC 562 (NR 025678.1) | 100 | Tepidibacter thalassicus strain SC 562 (NR 025678.1) | 27 | 0.83% |
| Sphingomonas phyllosphaerae | 0.8% | Sphingomonas phyllosphaerae strain FA2 (NR 029111.1) | 100 | Sphingomonas phyllosphaerae strain FA2 (NR 029111.1) | 14 | 0.43% |
| Microbacteriaceae_sp | 0.8% | Frondihabitans australicus strain E1HC-02 (NR 043897.1) | 100 | Frondihabitans australicus strain E1HC-02 (NR 043897.1) | 38 | 1.17% |
| Methylophilus_sp | 0.7% | Methylophilus flavus strain Ship (NR 104519.1) | 100 | Methylophilus flavus strain Ship (NR 104519.1) | 16 | 0.49% |
| Comamonadaceae_sp | 0.7% | Piscinibacter aquaticus strain IMCC1728 (NR 043921.1) | 100 | Piscinibacter aquaticus strain IMCC1728 (NR 043921.1) | 10 | 0.43% |
| Phycicoccus_sp | 0.7% | Phycicoccus dokdonensis strain DS-8 (NR 044286.1) | 100 | Phycicoccus dokdonensis strain DS-8 (NR 044286.1) | 9 | 0.28% |
| Legionella maceachernii | 0.5% | Legionella maceachernii strain ATCC 35300 (NR 041790.1) | 100 | Legionella maceachernii strain ATCC 35300 (NR 041790.1) | 22 | 0.68% |
| Comamonadaceae_sp | 0.5% | Variovorax paradoxus strain 13-0-1D (NR 036930.1) | 100 | Variovorax paradoxus strain 13-0-1D (NR 036930.1) | 9 | 0.28% |
| Caloranaerobacter azorensis | 0.5% | Caloranaerobacter azorensis strain MV1087 (NR 028919.1) | 73 | *incomplete WGS coverage of region* |  |  |
| Clostridium grantii | 0.4% | Clostridium grantii strain A1 (NR 026131.1) | 94 | Clostridium grantii strain A1 (NR 026131.1) | 6 | 0.19% |
| Bacillus coagulans | 0.4% | Bacillus coagulans strain NBRC 12583 (NR 041523.1) | 90 | Bacillus coagulans strain NBRC 12583 (NR 041523.1) | 17 | 0.52% |
| Promicromonospora umidemergens | 0.4% | Promicromonospora vindobonensis strain V-45 (NR 042146.1) | 73 | Promicromonospora vindobonensis strain V-45 (NR 042146.1) | 35 | 1.08% |
| Ferrithrix thermotolerans | 0.4% | Ferrithrix thermotolerans strain Y005 (NR 042751.1) | 14 | *incomplete WGS coverage of region* |  |  |
| Nocardia_g | 0.4% | Nocardia coubleae strain OFN N12 (NR 104567.1) | 100 | Nocardia coubleae strain OFN N12 (NR 104567.1) | 7 | 0.22% |
| Paracoccus pantotrophus | 0.4% | Paracoccus versutus strain ATCC 25364 (NR 042713.1) | 100 | Paracoccus versutus strain ATCC 25364 (NR 042713.1) | 22 | 0.68% |
| Pedobacter_sp | 0.3% | Pedobacter jejuensis strain THG-DR3 (NR 133810.1) | 84 | Pedobacter jejuensis strain THG-DR3 (NR 133810.1) | 10 | 0.31% |
| Kibdelosporangium aridum | 0.3% | Kibdelosporangium aridum subsp. largum strain SKF-AAD-609 (NR 025571.1) | 100 | Kibdelosporangium aridum subsp. largum strain SKF-AAD-609 (NR 025571.1) | 13 | 0.40% |
| Acholeplasma oculi | 0.3% | Acholeplasma oculi strain 19L (NR 025960.1) | 11 | *incomplete WGS coverage of region* |  |  |
| Nitrosomonas europaea | 0.3% | Nitrosomonas europaea strain C-31 (NR 040879.1) | 96 | Nitrosomonas europaea strain C-31 (NR 040879.1) | 18 | 0.56% |
| Pedobacter agri | 0.3% | Pedobacter ginsenosidimutans strain THG-45 (NR 108685.1) | 100 | Pedobacter ginsenosidimutans strain THG-45 (NR 108685.1) | 1 | 0.03% |
| Clostridium tetani | 0.3% | Clostridium cochlearium strain ATCC 17787 (NR 044717.2) | 54 | *incomplete WGS coverage of region* |  |  |
| Caminicella sporogenes | 0.3% | Caminicella sporogenes strain AM1114 (NR 025485.1) | 100 | *incomplete WGS coverage of region* |  |  |
| Butyrivibrio fibrisolvens | 0.2% | Butyrivibrio fibrisolvens strain ATCC 19171 (NR 025981.1) | 99 | Butyrivibrio fibrisolvens strain ATCC 19171 (NR 025981.1) | 9 | 0.28% |
| Tessaracoccus bendigoensis | 0.2% | Tessaracoccus flavescens strain SST-39 (NR 042550.1) | 48 | *incomplete WGS coverage of region* |  |  |
| Porphyromonas crevioricanis | 0.2% | Porphyromonas crevioricanis strain NUM 402 (NR 104834.1) | 34 | *incomplete WGS coverage of region* |  |  |
| Phenylobacterium composti | 0.2% | Phenylobacterium zucineum strain HLK1 (NR 074119.1) | 100 | Phenylobacterium zucineum strain HLK1 (NR 074119.1) | 6 | 0.19% |
| Curtobacterium sp. B20 | 0.2% | Curtobacterium flaccumfaciens strain LMG 3645 (NR 025467.1) | 81 | Curtobacterium flaccumfaciens strain LMG 3645 (NR 025467.1) | 5 | 0.15% |
| Butyrivibrio hungatei | 0.2% | Butyrivibrio hungatei strain JK 615 (NR 025525.1) | 100 | Butyrivibrio hungatei strain JK 615 (NR 025525.1) | 7 | 0.22% |
| Xanthobacter autotrophicus | 0.2% | Xanthobacter autotrophicus strain 7c (NR 026308.1) | 93 | Xanthobacter autotrophicus strain 7c (NR 026308.1) | 7 | 0.22% |
| Mycobacterium_sp | 0.2% | Mycobacterium obuense strain 47001 (NR 029218.1) | 29 | *incomplete WGS coverage of region* |  |  |
| [Eubacterium] yurii | 0.1% | [Eubacterium] yurii strain SM14 (NR 104843.1) | 100 | *incomplete WGS coverage of region* |  |  |
| Pseudoxanthobacter soli | 0.1% | Pseudoxanthobacter soli strain CC4 (NR 044225.1) | 63 | *incomplete WGS coverage of region* |  |  |
| Ohtaekwangia koreensis | 0.1% | Ohtaekwangia koreensis strain 3B-2 (NR 117435.1) | 70 | *incomplete WGS coverage of region* |  |  |
| Rhodobaca barguzinensis | 0.1% | Rhodobaca barguzinensis strain VKM B-2406 (NR 044285.1) | 92 | Rhodobaca barguzinensis strain VKM B-2406 (NR 044285.1) | 5 | 0.15% |
| Sphingobacterium nematocida | 0.1% | Sphingobacterium nematocida strain M-SX103 (NR 122101.1) | 34 | *incomplete WGS coverage of region* |  |  |
| Chryseobacterium_sp | 0.1% | Chryseobacterium gregarium strain P 461/12 (NR 042647.1) | 19 | *incomplete WGS coverage of region* |  |  |
| Lutispora thermophila | 0.0% | Lutispora thermophila strain EBR46 (NR 041236.1) | 54 | *incomplete WGS coverage of region* |  |  |
| Paenibacillus frigoriresistens | 0.0% | Paenibacillus frigoriresistens strain YIM 016 (NR 109546.1) | 36 | *incomplete WGS coverage of region* |  |  |
| Ohtaekwangia kribbensis | 0.0% | *Ohtaekwangia kribbensis strain 10AO (NR 117436.1)* | 83 | *incomplete WGS coverage of region* |  |  |
| Rhizobium_sp | 0.0% | Rhizobium daejeonense strain L61 (NR 042851.1) | 35 | *incomplete WGS coverage of region* |  |  |
| Prevotella oulorum | 0.0% | Prevotella oulorum strain WPH 179 (NR 029147.1) | 0 | *incomplete WGS coverage of region* |  |  |
| Alysiella filiformis | 0.0% | Alysiella crassa strain IAM 14969 (NR 040932.1) | 11 | *incomplete WGS coverage of region* |  |  |
| *Aquamicrobium_sp* | 0.0% | Mesorhizobium chacoense strain PR5 (NR 025411.1) | 65 | *incomplete WGS coverage of region* |  |  |
